# Supplementary material for: An issue of concern: unique truncated ORF8 protein variants of SARS-CoV-2
Source: PeerJ. 2022 Mar 21;10:e13136. doi: 10.7717/peerj.13136 (PMC8944340; doi:10.7717/peerj.13136)
Supplement: Supplemental Information 1 [file peerj-10-13136-s001.docx]

**Supplementary Materials**

An Issue of Concern: Unique Truncated ORF8 Protein Variants of SARS-CoV-2

**Sk. Sarif Hassan^1^, Vaishnavi Kodakandla^2^, Elrashdy M. Redwan^3^, Kenneth Lundstrom^4^, Pabitra Pal Choudhury^5^, Tarek Mohamed Abd El-Aziz^6^, Kazuo Takayama^7^, Ramesh Kandimalla^8^, Amos Lal^9^, A´ngel Serrano-Aroca^10^, Gajendra Kumar Azad^11^, Alaa A. A. Aljabali^12^, Giorgio Palu^13^, Gaurav Chauhan^14^, Parise Adadi^15^, Murtaza Tambuwala^16^, Adam M. Brufsky^17^, Wagner Baetas-da-Cruz^18^, Debmalya Barh^19^, Vasco Azevedo^20^, Nicolas G Bazan^21^, Bruno Silva Andrade^22^, Raner Jośe Santana Silva^23^, and Vladimir N. Uversky^24,*^**

*^1^ Department of Mathematics, Pingla Thana Mahavidyalaya, Maligram, Paschim Medinipur, 721140, West Bengal, India;*

*^2^ Department of Life sciences, Sophia College For Women, University of Mumbai, Bhulabhai Desai Road, Mumbai 400026, India;*

*^3^ Faculty of Science, Department of Biological Science, King Abdulaziz University, Jeddah 21589, Saudi Arabia;*

*^4^ PanTherapeutics, Rte de Lavaux 49, CH1095 Lutry, Switzerland;*

*^5^ Applied Statistics Unit, Indian Statistical Institute, 203 B T Road, Kolkata 700108, India;*

*^6^ Department of Cellular and Integrative Physiology, University of Texas Health Science Center at San Antonio, 7703 Floyd Curl Dr, San Antonio, TX 78229-3900, USA & Zoology Department, Faculty of Science, Minia University, El-Minia 61519, Egypt;*

*^7^ Center for iPS Cell Research and Application (CiRA), Kyoto University, Kyoto 6068507, Japan;*

*^8^ Applied Biology, CSIR-Indian Institute of Chemical Technology, Uppal Road, Tarnaka, Hyderabad, 500007, and Department of Biochemistry, Kakatiya Medical College, Warangal, Telangana, India;*

*^9^ Division of Pulmonary and Critical Care Medicine, Mayo Clinic, Rochester, Minnesota, USA;*

*^10^ Biomaterials and Bioengineering Lab, Centro de Investigacio´ n Traslacional San Alberto Magno, Universidad Cato´ lica de Valencia San Vicente Ma´rtir, c/Guillem de Castro, 94, 46001 Valencia, Valencia, Spain;*

*^11^ Department of Zoology, Patna University, Patna, Bihar, India;*

*^12^ Department of Pharmaceutics and Pharmaceutical Technology, Yarmouk University, Faculty of Pharmacy, Irbid 566, Jordan;*

*^13^ Department of Molecular Medicine, University of Padova, Via Gabelli 63, 35121, Padova, Italy;*

*^14^ School of Engineering and Sciences, Tecnologico de Monterrey, Av. Eugenio Garza Sada 2501 Sur, 64849 Monterrey, Nuevo Leo´ n, Mexico;*

*^15^ Department of Food Science, University of Otago, Dunedin 9054, New Zealand;*

*^16^ School of Pharmacy and Pharmaceutical Science, Ulster University, Coleraine BT52 1SA, Northern Ireland, UK;*

*^17^ University of Pittsburgh School of Medicine, Department of Medicine, Division of Hematology/Oncology, UPMC Hillman Cancer Center, Pittsburgh, PA, USA;*

*^18^ Translational Laboratory in Molecular Physiology, Centre for Experimental Surgery, College of Medicine, Federal University of Rio de Janeiro (UFRJ), Rio de Janeiro, Brazil;*

*^19^ Centre for Genomics and Applied Gene Technology, Institute of Integrative Omics and 46 Applied Biotechnology (IIOAB), Nonakuri, Purba Medinipur, West Bengal, India, and Departamento de Genetica, Ecologia e Evolucao, Instituto de Cieˆncias Biolo´ gicas, Universidade Federal de Minas Gerais, Belo Horizonte, Brazil;*

*^20^ Departamento de Genetica, Ecologia e Evolucao, Instituto de Cieˆncias Biolo´ gicas, Universidade Federal de Minas Gerais, Belo Horizonte, Brazil;*

*^21^ Neuroscience Center of Excellence, School of Medicine, LSU Health New Orleans, New Orleans, LA 70112, USA*

*^22^ Laboratório de Bioinformática e Química Computacional, Departamento de Ciências Biológicas, Universidade Estadual do Sudoeste da Bahia (UESB), Jequié, 45206-190, Brazil*

*^23^ Departamento de Ciencias Biologicas (DCB), Programa de Pos-Graduacao em Genetica e Biologia Molecular (PPGGBM), Universidade Estadual de Santa Cruz (UESC), Rodovia Ilheus-Itabuna, km 16, 45662-900, Ilheus, BA, Brazil*

*^24^ Department of Molecular Medicine, Morsani College of Medicine, University of South Florida, Tampa, FL 33612, USA*

** Corresponding author: Vladimir N. Uversky; Email address:* [*vuversky@usf.edu*](mailto:vuversky@usf.edu)

**
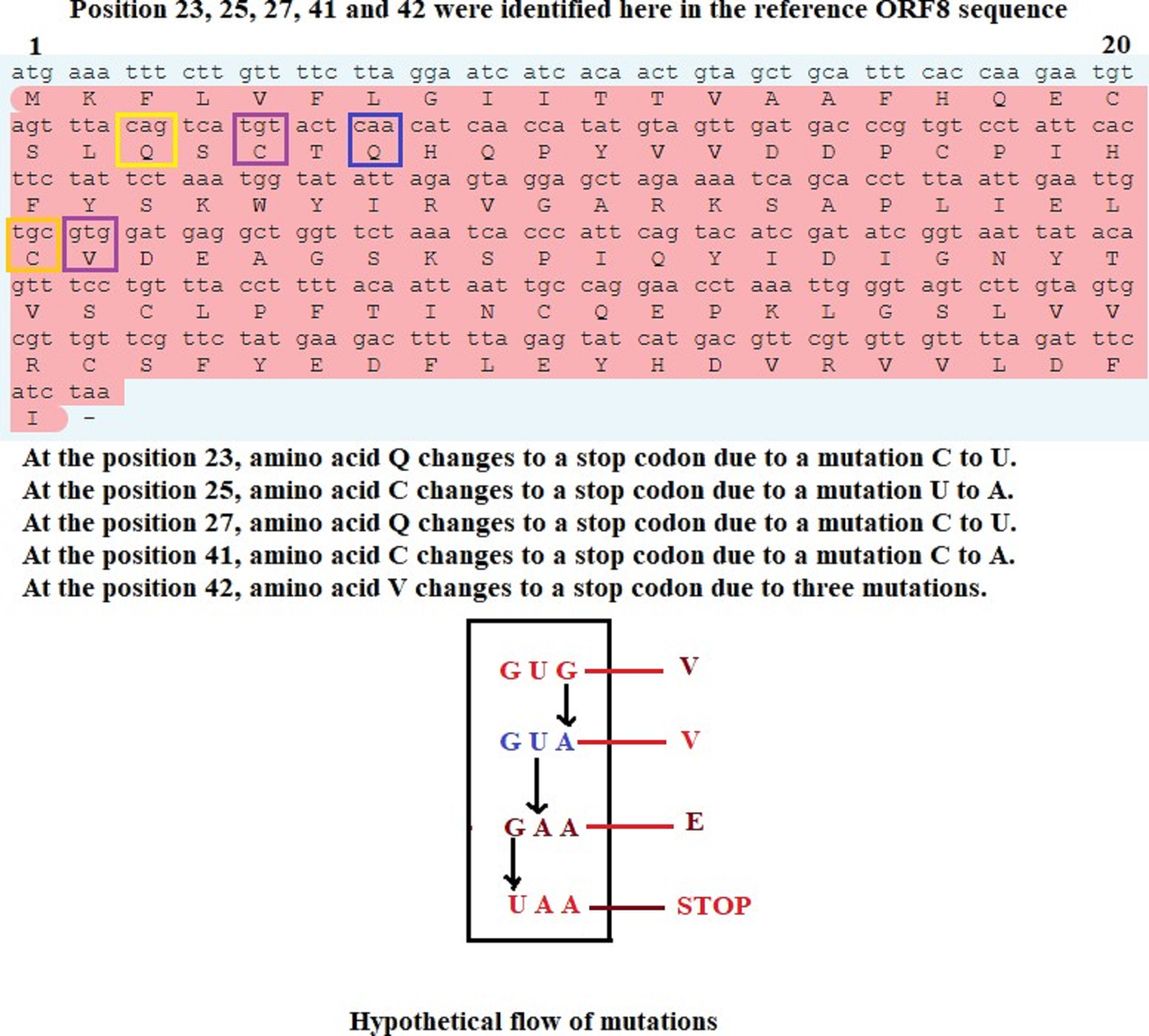
**

**Figure S1.** Possible mutations for truncation at 23, 25, 27, 40, and 42 residue positions of ORF8 protein (*NC* 045512) of SARS-CoV-2.


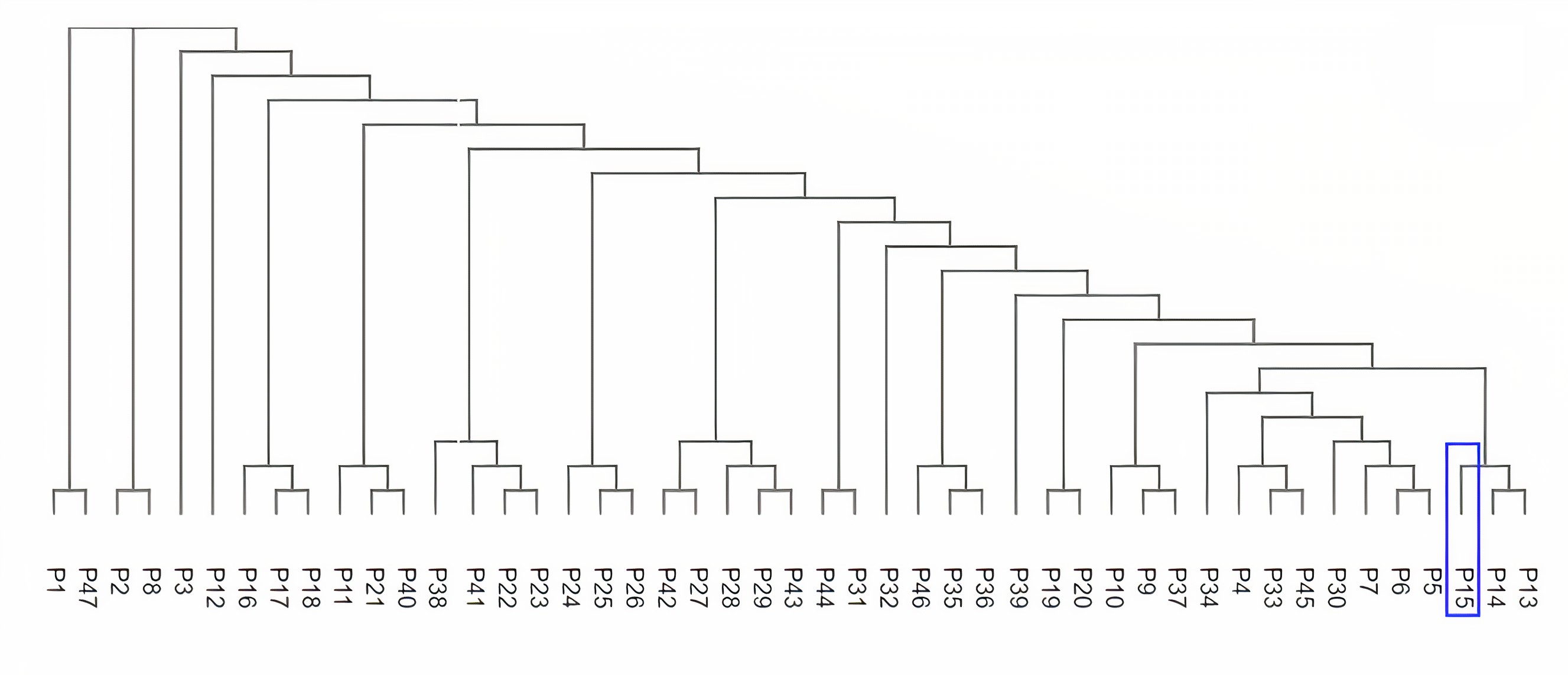
**Supplementary Figure S2.** Phylogenetic relationship among the unique T-ORF8 proteins.

**
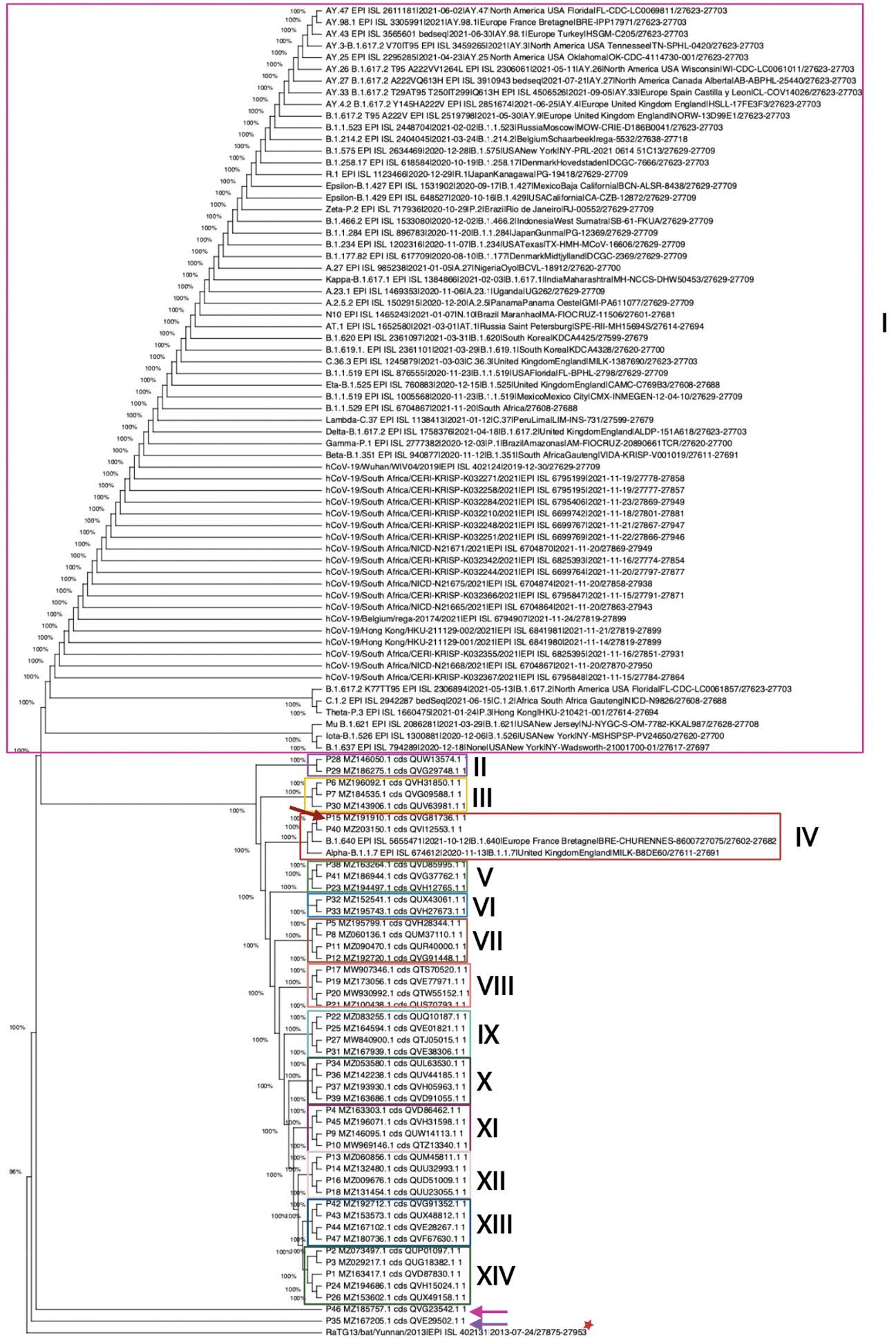
**

**Supplementary Figure S3.** Maximum Likelihood phylogenetic tree for the 47 truncated ORF8 and 66 representative ORF8 sequences for different Alpha, Beta, Gamma, Delta, Mu, GH/490R, and Omicron variants, using 500 bootstrap replications and the Hasegawa-Kishino-Yano model. 14 group clades were found and the sequences 15 and 40 are clearly forming a group clade with Alpha and B.1.640 ORF 8 sequences. Sequences 35 and 46 are indicated by marine blue and purple arrows, respectively and sequence 15 is indicated by a red arrow.


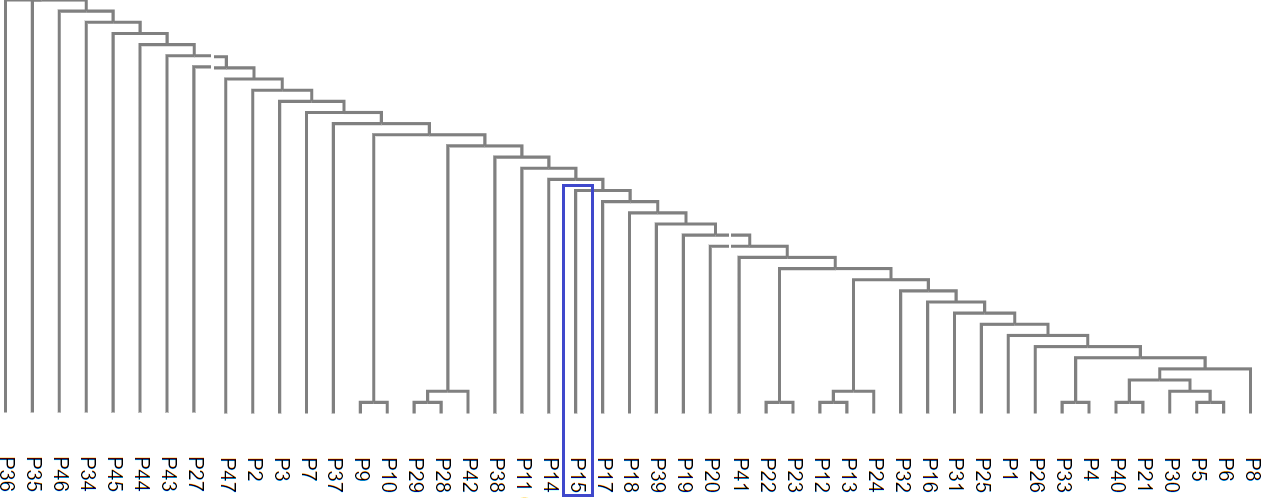


**Supplementary Figure S4.** Analysis of variability among unique T-ORF8 variants based on polarity: Polarity-based phylogenetic relationships among the unique T-ORF8 proteins.

**
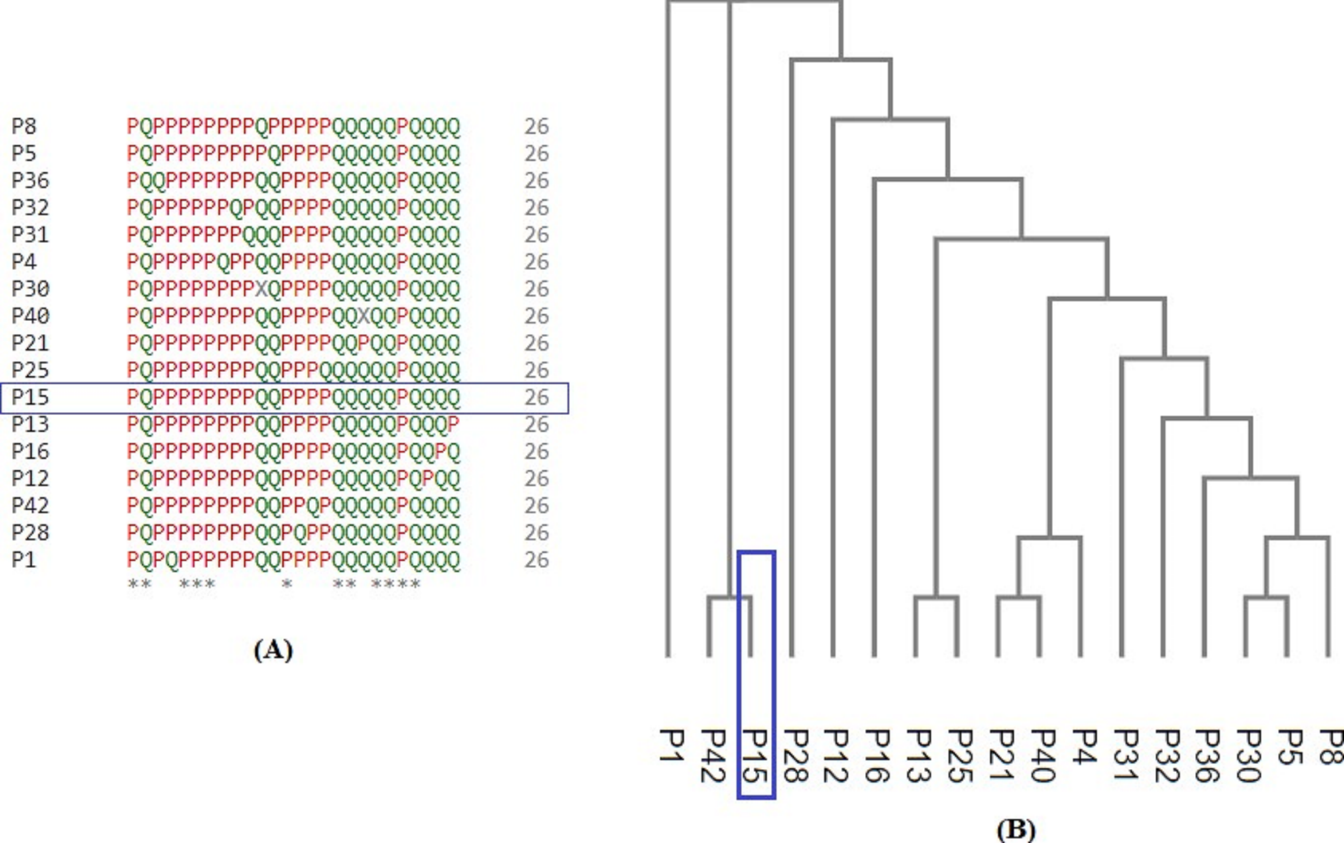
**

**Figure S5.** Unique polar/non-polar sequence-based alignment of unique T-ORF8 proteins using the Clustal-Omega and associated phylogenetic relationship. **A**. multiple sequence alignments of unique T-ORF8, and **B**. phylogenetic analysis of the same samples.

**
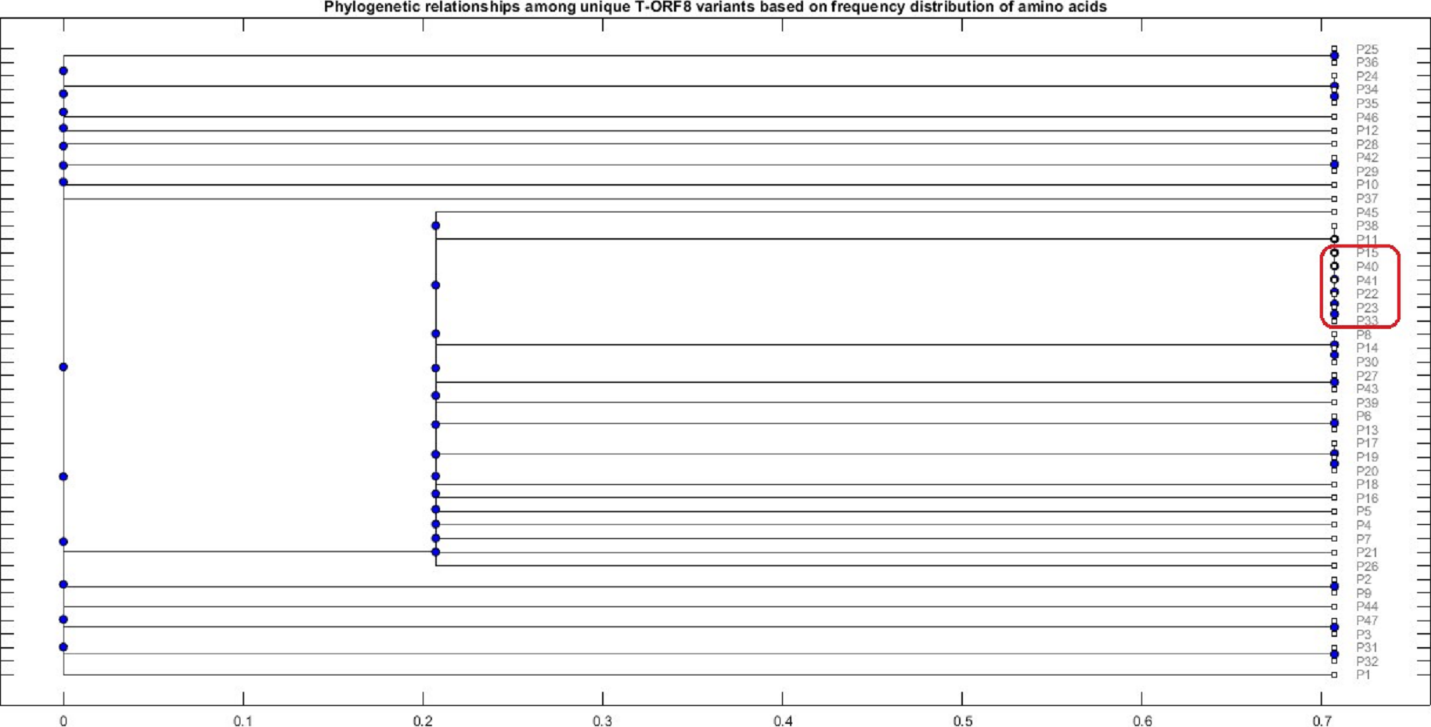
**

**Figure S6.** Frequency distribution of amino acids present in the unique T-ORF8 variants and their phylogenetic relationships. The red box highlights the closeness of P15 with P22, P23, P33, P40, and P41 variants.

**
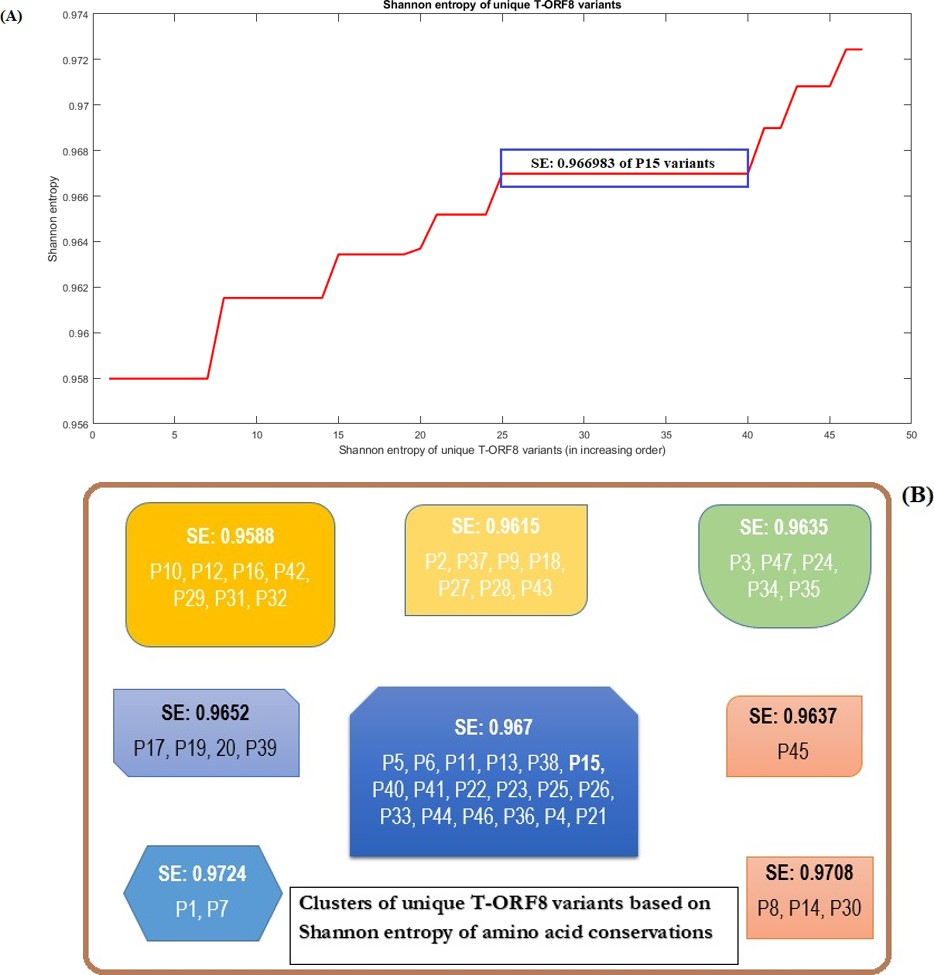
**

**Figure S7.** Analysis of T-ORF8 variability: **A**. Shannon entropy of unique T-ORF8 protein variants (plotted in increasing order), and **B**. Clusters of T-ORF8 variants based on Shannon entropy. The boxes highlighted in different colors represent clusters of unique T-ORF8 variants.

**Supplementary Table S1.** List of unique truncated ORF8 proteins and their representative accession IDs

| Unique variants of truncated ORF8 proteins (world-wide) | | |
| --- | --- | --- |
| ***Serial Name*** | ***Representative Accession ID*** | ***Unique T-ORF8 Sequence*** |
| **P1** | QVD87830.1 | MKFHVFLGIITTVAAFHQECSLQSCT |
| **P2** | QUP01097.1 | MKFLIFLGIITTVAAFHQECSLQSCT |
| **P3** | QUG18382.1 | MKFLVFFGIITTVAAFHQECSLQSCT |
| **P4** | QVD86462.1 | MKFLVFLEIITTVAAFHQECSLQSCT |
| **P5** | QVH28344.1 | MKFLVFLGIIATVAAFHQECSLQSCT |
| **P6** | QVH31850.1 | MKFLVFLGIIITVAAFHQECSLQSCT |
| **P7** | QVG09588.1 | MKFLVFLGIIKTVAAFHQECSLQSCT |
| **P8** | QUM37110.1 | MKFLVFLGIITPVAAFHQECSLQSCT |
| **P9** | QUW14113.1 | MKFLVFLGIITTIAAFHQECSLQSCT |
| **P10** | QTZ13340.1 | MKFLVFLGIITTLAAFHQECSLQSCT |
| **P11** | QUR40000.1 | MKFLVFLGIITTVAAFHQDCSLQSCT |
| **P12** | QVG91448.1 | MKFLVFLGIITTVAAFHQECSLQLCT |
| **P13** | QUM45811.1 | MKFLVFLGIITTVAAFHQECSLQSCI |
| **P14** | QUU32993.1 | MKFLVFLGIITTVAAFHQECSLQSCN |
| **P15** | QVG81736.1 | **MKFLVFLGIITTVAAFHQECSLQSCT** |
| **P16** | QUD51009.1 | MKFLVFLGIITTVAAFHQECSLQSFT |
| **P17** | QTS70520.1 | MKFLVFLGIITTVAAFHQECSLQSRT |
| **P18** | QUU23055.1 | MKFLVFLGIITTVAAFHQECSLQSST |
| **P19** | QVE77971.1 | MKFLVFLGIITTVAAFHQERSLQSCT |
| **P20** | QTW55152.1 | MKFLVFLGIITTVAAFHQEYSLQSCT |
| **P21** | QUS70793.1 | MKFLVFLGIITTVAAFHQGCSLQSCT |
| **P22** | QUQ10187.1 | MKFLVFLGIITTVAAFRQECSLQSCT |
| **P23** | QVH12765.1 | MKFLVFLGIITTVAAFYQECSLQSCT |
| **P24** | QVH15024.1 | MKFLVFLGIITTVAALHQECSLQSCT |
| **P25** | QVE01821.1 | MKFLVFLGIITTVAASHQECSLQSCT |
| **P26** | QUX49158.1 | MKFLVFLGIITTVAAVHQECSLQSCT |
| **P27** | QTJ05015.1 | MKFLVFLGIITTVAVFHQECSLQSCT |
| **P28** | QUW13574.1 | MKFLVFLGIITTVSAFHQECSLQSCT |
| **P29** | QVG29748.1 | MKFLVFLGIITTVTAFHQECSLQSCT |
| **P30** | QUV63981.1 | MKFLVFLGIIXTVAAFHQECSLQSCT |
| **P31** | QVE38306.1 | MKFLVFLGITTTVAAFHQECSLQSCT |
| **P32** | QUX43061.1 | MKFLVFLGTITTVAAFHQECSLQSCT |
| **P33** | QVH27673.1 | MKFLVFLRIITTVAAFHQECSLQSCT |
| **P34** | QUL63530.1 | MKFLVLLGIITTVAAFHQECSLQSCT |
| **P35** | QVE29502.1 | MKLLVFLGIITTVAAFHQECSLQSCT |
| **P36** | QUV44185.1 | MKSLVFLGIITTVAAFHQECSLQSCT |
| **P37** | QVH05963.1 | MKFLVFLGIITTAAAFHQECSLQSCT |
| **P38** | QVD85995.1 | MKFLVFLGIITTVAAFDQECSLQSCT |
| **P39** | QVD91055.1 | MKFLVFLGIITTVAAFHQECSLRSCT |
| **P40** | QVI12553.1 | MKFLVFLGIITTVAAFHQXCSLQSCT |
| **P41** | QVG37762.1 | MKFLVFLGIITTVAAFNQECSLQSCT |
| **P42** | QVG91352.1 | MKFLVFLGIITTVATFHQECSLQSCT |
| **P43** | QUX48812.1 | MKFLVFLGIITTVVAFHQECSLQSCT |
| **P44** | QVE28267.1 | MKFLVFLGIMTTVAAFHQECSLQSCT |
| **P45** | QVH31598.1 | MKFLVFLVIITTVAAFHQECSLQSCT |
| **P46** | QVG23542.1 | MKILVFLGIITTVAAFHQECSLQSCT |
| **P47** | QVF67630.1 | MKFFVFLGIITTVAAFHQECSLQSCT |

**Supplementary Table S2**: Collection date, geo-location, frequency of presence and accession ID of the first identified of each unique T-ORF8 variants

| **Unique T-ORF8** | **Frequency of presence** | **Accession id** | **Collection date** | **Geo-location** |
| --- | --- | --- | --- | --- |
| P1 | 2 | QUI86380.1 | 06-04-2021 | USA: California |
| P2 | 13 | QTY80195.1 | 13-03-2021 | USA: Ohio |
| P3 | 1 | QUG18382.1 | 02-04-2021 | USA: Minnesota |
| P4 | 7 | QSU72470.1 | 12-02-2021 | USA: Florida |
| P5 | 7 | QTY89054.1 | 18-03-2021 | USA: Tennessee |
| P6 | 18 | QTF76874.1 | 20-02-2021 | USA: Texas |
| P7 | 3 | QUM35363.1 | 07-04-2021 | USA: Pennsylvania |
| P8 | 1 | QUM37110.1 | 08-04-2021 | USA: Michigan |
| P9 | 1 | QUW14113.1 | 19-04-2021 | USA: Texas |
| P10 | 1 | QTZ13340.1 | 23-03-2021 | USA: New Jersey |
| P11 | 1 | QUR40000.1 | 14-04-2021 | USA: Texas |
| P12 | 2 | QUP81732.1 | 16-04-2021 | USA: Minnesota |
| P13 | 3 | QUE25142.1 | 29-03-2021 | USA: Minnesota |
| P14 | 1 | QUU32993.1 | 16-04-2021 | USA: Florida |
| **P15** |  | **QUJ17746** | **15-03-2020** | **Europe: Poland** |
| **P15** |  | **QUJ17770** | **15-03-2020** | **Europe: Poland** |
| **P15** |  | **QQH16621** | **31-05-2020** | **Asia: Pakistan-Punjab** |
| **P15** | **48395** | **QRN78390** | **10-12-2020** | **Africa: Ghana** |
| **P15** |  | **QQV29253** | **31-12-2020** | **South America: Peru** |
| **P15** |  | **QMU25282** | **27-05-2020** | **USA: Maryland** |
| **P15** |  | **QMU25294** | **27-05-2020** | **USA: Maryland** |
| P16 | 23 | QTG22339.1 | 21-02-2021 | USA: Florida |
| P17 | 1 | QTS70520.1 | 16-03-2021 | USA: Pennsylvania |
| P18 | 3 | QUC96581.1 | 29-03-2021 | USA: Illinois |
| P19 | 4 | QUC99721.1 | 29-03-2021 | USA: Pennsylvania |
| P20 | 1 | QTW55152.1 | 27-03-2021 | USA: Pennsylvania |
| P21 | 1 | QUS70793.1 | 15-04-2021 | USA: Georgia |
| P22 | 1 | QUQ10187.1 | 06-04-2021 | USA: Tennessee |
| P23 | 24 | QTJ05327.1 | 03-03-2021 | USA: Pennsylvania |
| P24 |  | QUQ10379.1 | 05-04-2021 | USA: Puerto Rico |
| P24 | 28 | QUQ37029.1 | 05-04-2021 | USA: Puerto Rico |
| P24 |  | QUA75771.1 | 05-04-2021 | USA: Puerto Rico |
| P25 | 16 | QTX01933.1 | 11-03-2021 | USA: Texas |
| P26 | 2 | QUQ28684.1 | 08-04-2021 | USA: Maryland |
| P27 | 1 | QTJ05015.1 | 02-03-2021 | USA: Pennsylvania |
| P28 | 2 | QUQ51956.1 | 07-04-2021 | USA: Missouri |
| P29 | 19 | QTZ09174.1 | 26-03-2021 | USA: Maryland |
| P30 | 1 | QUV63981.1 | 11-04-2021 | USA: California |
| P31 | 7 | QTZ05620.1 | 25-03-2021 | USA: Louisiana |
| P32 | 5 | QTM88238.1 | 09-03-2021 | USA: Connecticut |

**Supplementary Table S3**. Results of the ELM search for functional motifs in 47 T-ORF8 proteins

| T-ORF8 | Elm name | Instances | Position | ELM description | Cell compartment |
| --- | --- | --- | --- | --- | --- |
| P1, P5, P6, P7, P9, P10, P11, P14, P15, P22, P23, P24, P26, P30, P32, P34, P35, P37, P38, P41, P44, P46,  P47 | DEG_Nend_UBRbox_1 | MKFH | 1-4 | N-terminal motif that initiates protein degradation by binding to the UBR-box of N-recognins. This N-degron variant comprises N-terminal Arg or Lys as destabilizing residue | cytosol |
|  | DOC_MAPK_MEF2A_6 | KFHVFLGI | 2-9 | A kinase docking motif that mediates interaction towards the ERK1/2 and p38 subfamilies of MAP kinases | nucleus, cytosol, Transcription factor complex |
|  | MOD_OFUCOSY | CSLQSC | 20-25 | Site for attachment of a fucose residue to a serine | extracellular |
|  | MOD_Plk_1 | QECSLQS | 18-24 | Ser/Thr residue phosphorylated by the Plk1 kinase | centralspindlin complex, nucleus,  spindle, gamma-tubulin complex,  midbody, cytosol, kinetochore,  spindle midzone, nuclear condensin complex, cleavage furrow, nucleoplasm, microtubule organizing center |
| P12, P16, P17, P19 | DEG_Nend_UBRbox_1 | MKFH | 1-4 | N-terminal motif that initiates protein degradation by binding to the UBR-box of N-recognins. This N-degron variant comprises N-terminal Arg or Lys as destabilizing residue | cytosol |
|  | DOC_MAPK_MEF2A_6 | KFHVFLGI | 2-9 | A kinase docking motif that mediates interaction towards the ERK1/2 and p38 subfamilies of MAP kinases | nucleus, cytosol, Transcription factor complex |
|  | MOD_Plk_1 | QECSLQS | 18-24 | Ser/Thr residue phosphorylated by the Plk1 kinase | centralspindlin complex, nucleus,  spindle, gamma-tubulin complex,  midbody, cytosol, kinetochore,  spindle midzone, nuclear condensin complex, cleavage furrow, nucleoplasm, microtubule organizing center |
| P21, P40 | DEG_Nend_UBRbox_1 | MKFH | 1-4 | N-terminal motif that initiates protein degradation by binding to the UBR-box of N-recognins. This N-degron variant comprises N-terminal Arg or Lys as destabilizing residue | cytosol |
|  | DOC_MAPK_MEF2A_6 | KFHVFLGI | 2-9 | A kinase docking motif that mediates interaction towards the ERK1/2 and p38 subfamilies of MAP kinases | nucleus, cytosol, Transcription factor complex |
|  | MOD_OFUCOSY | CSLQSC | 20-25 | Site for attachment of a fucose residue to a serine. | extracellular |
| P2 | DEG_Nend_UBRbox_1 | MKFH | 1-4 | N-terminal motif that initiates protein degradation by binding to the UBR-box of N-recognins. This N-degron variant comprises N-terminal Arg or Lys as destabilizing residue | cytosol |
|  | DOC_MAPK_MEF2A_6 | KFHVFLGI | 2-9 | A kinase docking motif that mediates interaction towards the ERK1/2 and p38 subfamilies of MAP kinases | nucleus, cytosol, Transcription factor complex |
|  | DOC_MAPK_NFAT4_5 | KFLIFLGII | 2-10 | An extended D site specifically recognized by the JNK kinases | cytosol, nucleus, protein complex, neuron projection |
|  | MOD_OFUCOSY | CSLQSC | 20-25 | Site for attachment of a fucose residue to a serine | extracellular |
|  | MOD_Plk_1 | QECSLQS | 18-24 | Ser/Thr residue phosphorylated by the Plk1 kinase | centralspindlin complex, nucleus, spindle, gamma-tubulin complex, midbody, cytosol, kinetochore, spindle midzone, nuclear condensin complex, cleavage furrow, nucleoplasm, microtubule organizing center |
| P3 | DEG_Nend_UBRbox_1 | MKFH | 1-4 | N-terminal motif that initiates protein degradation by binding to the UBR-box of N-recognins. This N-degron variant comprises N-terminal Arg or Lys as destabilizing residue | cytosol |
|  | LIG_Pex14_2 | FLVFF | 3-7 | Fxxx[WF] motifs are present in Pex19 and S. cerevisiae Pex5 cytosolic receptors that bind to peroxisomal membrane docking member, Pex14 | cytosol, peroxisome, glycosome |
|  | MOD_OFUCOSY | CSLQSC | 20-25 | Site for attachment of a fucose residue to a serine | extracellular |
|  | MOD_Plk_1 | QECSLQS | 18-24 | Ser/Thr residue phosphorylated by the Plk1 kinase | centralspindlin complex, nucleus,  spindle, gamma-tubulin complex,  midbody, cytosol, kinetochore,  spindle midzone, nuclear condensin complex, cleavage furrow, nucleoplasm, microtubule organizing center |
| P4 | DEG_Nend_UBRbox_1 | MKFL | 1-4 | N-terminal motif that initiates protein degradation by binding to the UBR-box of N-recognins. This N-degron variant comprises N-terminal Arg or Lys as destabilizing residue | cytosol |
|  | DOC_MAPK_MEF2A_6 | KFLVFLEI | 2-9 | A kinase docking motif that mediates interaction towards the ERK1/2 and p38 subfamilies of MAP kinases | nucleus, cytosol, Transcription factor complex |
|  | LIG_APCC_ABBA_1 | FLVFLE | 3-8 | Amphipathic motif that is involved in APC/C inhibition by binding of CDH1/CDC20. In metazoan cyclin A, the motif also acts as a degron, enabling the cyclin's degradation in prometaphase. | spindle pole, nucleus,  cytosol |
|  | LIG_APCC_ABBAyCdc20_2 | KFLVFLE | 2-8 | Amphipathic motif that binds to yeast Cdc20 and acts as an APC/C degron enabling cyclin Clb5 degradation during mitosis. | not annotated |
|  | LIG_Clathr_ClatBox_1 | LVFLE | 4-8 | Clathrin box motif found on cargo adaptor proteins, it interacts with the beta propeller structure located at the N-terminus of Clathrin heavy chain. | cytosol, Golgi apparatus,  cytoskeleton, clathrin-coated endocytic vesicle,  Golgi trans-face |
|  | MOD_OFUCOSY | CSLQSC | 20-25 | Site for attachment of a fucose residue to a serine | extracellular |
|  | MOD_Plk_1 | QECSLQS | 18-24 | Ser/Thr residue phosphorylated by the Plk1 kinase | centralspindlin complex, nucleus,  spindle, gamma-tubulin complex,  midbody, cytosol, kinetochore,  spindle midzone, nuclear condensin complex, cleavage furrow, nucleoplasm, microtubule organizing center |
| P8 | DEG_Nend_UBRbox_1 | MKFL | 1-4 | N-terminal motif that initiates protein degradation by binding to the UBR-box of N-recognins. This N-degron variant comprises N-terminal Arg or Lys as destabilizing residue | cytosol |
|  | DOC_CKS1_1 | IITPVA | 9-14 | Phospho-dependent motif that mediates docking of CDK substrates and regulators to cyclin-CDK-bound Cks1. | cytosol, nucleus |
|  | DOC_MAPK_MEF2A_6 | KFLVFLEI | 2-9 | A kinase docking motif that mediates interaction towards the ERK1/2 and p38 subfamilies of MAP kinases | nucleus, cytosol, Transcription factor complex |
|  | DOC_WW_Pin1_4 | GIITPV | 8-13 | The Class IV WW domain interaction motif is recognised primarily by the Pin1 phosphorylation-dependent prolyl isomerase. | cytosol, nucleus |
|  | MOD_OFUCOSY | CSLQSC | 20-25 | Site for attachment of a fucose residue to a serine | extracellular |
|  | MOD_Plk_1 | QECSLQS | 18-24 | Ser/Thr residue phosphorylated by the Plk1 kinase | centralspindlin complex, nucleus,  spindle, gamma-tubulin complex,  midbody, cytosol, kinetochore,  spindle midzone, nuclear condensin complex, cleavage furrow, nucleoplasm, microtubule organizing center |
|  | MOD_ProDKin_1 | GIITPVA | 8-14 | Proline-Directed Kinase (e.g. MAPK) phosphorylation site in higher eukaryotes. | cytosol, nucleus |
| P13 | DEG_Nend_UBRbox_1 | MKFH | 1-4 | N-terminal motif that initiates protein degradation by binding to the UBR-box of N-recognins. This N-degron variant comprises N-terminal Arg or Lys as destabilizing residue | cytosol |
|  | DOC_MAPK_MEF2A_6 | KFHVFLGI | 2-9 | A kinase docking motif that mediates interaction towards the ERK1/2 and p38 subfamilies of MAP kinases | nucleus, cytosol, Transcription factor complex |
|  | LIG_PDZ_Class_1 | SLQSCI | 21-26 | The C-terminal class 1 PDZ-binding motif is classically represented by a pattern like (ST)X(VIL)* | cytosol, internal side of plasma membrane |
|  | MOD_OFUCOSY | CSLQSC | 20-25 | Site for attachment of a fucose residue to a serine | extracellular |
|  | MOD_Plk_1 | QECSLQS | 18-24 | Ser/Thr residue phosphorylated by the Plk1 kinase | centralspindlin complex, nucleus, spindle, gamma-tubulin complex, midbody, cytosol, kinetochore, spindle midzone, nuclear condensin complex, cleavage furrow, nucleoplasm, microtubule organizing center |
| P18 | DEG_Nend_UBRbox_1 | MKFH | 1-4 | N-terminal motif that initiates protein degradation by binding to the UBR-box of N-recognins. This N-degron variant comprises N-terminal Arg or Lys as destabilizing residue | cytosol |
|  | DOC_MAPK_MEF2A_6 | KFHVFLGI | 2-9 | A kinase docking motif that mediates interaction towards the ERK1/2 and p38 subfamilies of MAP kinases | nucleus, cytosol, Transcription factor complex |
|  | MOD_GSK3_1 | QECSLQSS | 18-25 | GSK3 phosphorylation recognition site | cytosol, nucleus |
|  | MOD_Plk_1 | QECSLQS | 18-24 | Ser/Thr residue phosphorylated by the Plk1 kinase | centralspindlin complex, nucleus,  spindle, gamma-tubulin complex,  midbody, cytosol, kinetochore,  spindle midzone, nuclear condensin complex, cleavage furrow, nucleoplasm, microtubule organizing center |
| P20 | DEG_Nend_UBRbox_1 | MKFH | 1-4 | N-terminal motif that initiates protein degradation by binding to the UBR-box of N-recognins. This N-degron variant comprises N-terminal Arg or Lys as destabilizing residue | cytosol |
|  | DOC_MAPK_MEF2A_6 | KFHVFLGI | 2-9 | A kinase docking motif that mediates interaction towards the ERK1/2 and p38 subfamilies of MAP kinases | nucleus, cytosol, Transcription factor complex |
|  | LIG_SH2_STAT3 | YSLQ | 20-23 | YXXQ motif found in the cytoplasmic region of cytokine receptors that bind STAT3 SH2 domain. | cytosol |
|  | MOD_Plk_1 | QECSLQS | 18-24 | Ser/Thr residue phosphorylated by the Plk1 kinase | centralspindlin complex, nucleus,  spindle, gamma-tubulin complex,  midbody, cytosol, kinetochore,  spindle midzone, nuclear condensin complex, cleavage furrow, nucleoplasm, microtubule organizing center |
| P25 | DEG_Nend_UBRbox_1 | MKFL | 1-4 | N-terminal motif that initiates protein degradation by binding to the UBR-box of N-recognins. This N-degron variant comprises N-terminal Arg or Lys as destabilizing residue | cytosol |
|  | DOC_MAPK_MEF2A_6 | KFLVFLGI | 2-9 | A kinase docking motif that mediates interaction towards the ERK1/2 and p38 subfamilies of MAP kinases | nucleus, cytosol, Transcription factor complex |
|  | LIG_TRAF2_1 | SHQE | 16-19 | Major TRAF2-binding consensus motif. Members of the tumor necrosis factor receptor (TNFR) superfamily initiate intracellular signaling by recruiting the C-domain of the TNFR-associated factors (TRAFs) through their cytoplasmic tails | cytosol |
|  | MOD_CK2_1 | VAASHQE | 13-19 | Casein kinase 2 (CK2) phosphorylation site | protein kinase CK2 complex,  nucleus, cytosol |
|  | MOD_GSK3_1 | IITTVAAS | 9-16 | GSK3 phosphorylation recognition site | cytosol, nucleus |
|  | MOD_OFUCOSY | CSLQSC | 20-25 | Site for attachment of a fucose residue to a serine. | extracellular |
|  | MOD_Plk_1 | QECSLQS | 18-24 | Ser/Thr residue phosphorylated by the Plk1 kinase | centralspindlin complex, nucleus, spindle, gamma-tubulin complex, midbody, cytosol, kinetochore, spindle midzone, nuclear condensin complex, cleavage furrow, nucleoplasm, microtubule organizing center |
| P27 | DEG_Nend_UBRbox_1 | MKFH | 1-4 | N-terminal motif that initiates protein degradation by binding to the UBR-box of N-recognins. This N-degron variant comprises N-terminal Arg or Lys as destabilizing residue | cytosol |
|  | DOC_MAPK_MEF2A_6 | KFHVFLGI | 2-9 | A kinase docking motif that mediates interaction towards the ERK1/2 and p38 subfamilies of MAP kinases | nucleus, cytosol, Transcription factor complex |
|  | LIG_FHA_1 | ITTVAVF | 10-16 | Phosphothreonine motif binding a subset of FHA domains that show a preference for a large aliphatic amino acid at the pT+3 position. | nucleus |
|  | MOD_OFUCOSY | CSLQSC | 20-25 | Site for attachment of a fucose residue to a serine | extracellular |
|  | MOD_Plk_1 | QECSLQS | 18-24 | Ser/Thr residue phosphorylated by the Plk1 kinase | centralspindlin complex, nucleus,  spindle, gamma-tubulin complex,  midbody, cytosol, kinetochore,  spindle midzone, nuclear condensin complex, cleavage furrow, nucleoplasm, microtubule organizing center |
| P28 | DEG_Nend_UBRbox_1 | MKFL | 1-4 | N-terminal motif that initiates protein degradation by binding to the UBR-box of N-recognins. This N-degron variant comprises N-terminal Arg or Lys as destabilizing residue | cytosol |
|  | DOC_MAPK_MEF2A_6 | KFLVFLGI | 2-9 | A kinase docking motif that mediates interaction towards the ERK1/2 and p38 subfamilies of MAP kinases | nucleus, cytosol, Transcription factor complex |
|  | LIG_WD40_WDR5_VDV_2 | TTVSA  TVSA | 11-15  12-15 | Fungi-specific variant of the WDR5-binding motif that binds to a cleft between blades 5 and 6 of the WD40 repeat domain of WDR5, opposite of the Win motif-binding site, to mediate assembly of histone modification complexes. | nucleus, histone methyltransferase complex |
|  | MOD_GlcNHglycan | VSAF | 13-16 | Glycosaminoglycan attachment site | extracellular,  Golgi apparatus |
|  | MOD_OFUCOSY | CSLQSC | 20-25 | Site for attachment of a fucose residue to a serine. | extracellular |
|  | MOD_Plk_1 | QECSLQS | 18-24 | Ser/Thr residue phosphorylated by the Plk1 kinase | centralspindlin complex, nucleus, spindle, gamma-tubulin complex, midbody, cytosol, kinetochore, spindle midzone, nuclear condensin complex, cleavage furrow, nucleoplasm, microtubule organizing center |
| P29 | DEG_Nend_UBRbox_1 | MKFL | 1-4 | N-terminal motif that initiates protein degradation by binding to the UBR-box of N-recognins. This N-degron variant comprises N-terminal Arg or Lys as destabilizing residue | cytosol |
|  | DOC_MAPK_MEF2A_6 | KFLVFLGI | 2-9 | A kinase docking motif that mediates interaction towards the ERK1/2 and p38 subfamilies of MAP kinases | nucleus, cytosol, Transcription factor complex |
|  | LIG_WD40_WDR5_VDV_2 | TTVTA  TVTA | 11-15  12-15 | Fungi-specific variant of the WDR5-binding motif that binds to a cleft between blades 5 and 6 of the WD40 repeat domain of WDR5, opposite of the Win motif-binding site, to mediate assembly of histone modification complexes. | nucleus, histone methyltransferase complex |
|  | MOD_OFUCOSY | CSLQSC | 20-25 | Site for attachment of a fucose residue to a serine. | extracellular |
|  | MOD_Plk_1 | QECSLQS | 18-24 | Ser/Thr residue phosphorylated by the Plk1 kinase | centralspindlin complex, nucleus, spindle, gamma-tubulin complex, midbody, cytosol, kinetochore, spindle midzone, nuclear condensin complex, cleavage furrow, nucleoplasm, microtubule organizing center |
| P31 | DEG_Nend_UBRbox_1 | MKFL | 1-4 | N-terminal motif that initiates protein degradation by binding to the UBR-box of N-recognins. This N-degron variant comprises N-terminal Arg or Lys as destabilizing residue | cytosol |
|  | DOC_MAPK_MEF2A_6 | KFLVFLGI | 2-9 | A kinase docking motif that mediates interaction towards the ERK1/2 and p38 subfamilies of MAP kinases | nucleus, cytosol, Transcription factor complex |
|  | LIG_FHA_1 | GITTTVA | 8-14 | Phosphothreonine motif binding a subset of FHA domains that show a preference for a large aliphatic amino acid at the pT+3 position. | nucleus |
|  | MOD_NEK2_1 | LGITTT | 7-12 | NEK2 phosphorylation motif with preferred Phe, Leu or Met in the -3 position to compensate for less favorable residues in the +1 and +2 position. | centrosome, Ndc80 complex, condensed nuclear chromosome outer kinetochore, cytosol, nucleus |
|  | MOD_OFUCOSY | CSLQSC | 20-25 | Site for attachment of a fucose residue to a serine. | extracellular |
|  | MOD_Plk_1 | QECSLQS | 18-24 | Ser/Thr residue phosphorylated by the Plk1 kinase | centralspindlin complex, nucleus, spindle, gamma-tubulin complex, midbody, cytosol, kinetochore, spindle midzone, nuclear condensin complex, cleavage furrow, nucleoplasm, microtubule organizing center |
| P33 | CLV_PCSK_SKI1_1 | RIITT | 8-12 | Subtilisin/kexin isozyme-1 (SKI1) cleavage site ([RK]-X-[hydrophobic]-[LTKF]-\|-X). | endoplasmic reticulum lumen, endoplasmic reticulum, Golgi apparatus,  extracellular |
|  | DEG_Nend_UBRbox_1 | MKFL | 1-4 | N-terminal motif that initiates protein degradation by binding to the UBR-box of N-recognins. This N-degron variant comprises N-terminal Arg or Lys as destabilizing residue | cytosol |
|  | DOC_MAPK_MEF2A_6 | KFLVFLGI | 2-9 | A kinase docking motif that mediates interaction towards the ERK1/2 and p38 subfamilies of MAP kinases | nucleus, cytosol, Transcription factor complex |
|  | LIG_14-3-3_CanoR_1 | RIITTVA | 8-14 | Canonical Arg-containing phospho-motif mediating a strong interaction with 14-3-3 proteins. | Nucleus, internal side of plasma membrane,  cytosol |
|  | MOD_OFUCOSY | CSLQSC | 20-25 | Site for attachment of a fucose residue to a serine. | extracellular |
|  | MOD_Plk_1 | QECSLQS | 18-24 | Ser/Thr residue phosphorylated by the Plk1 kinase | centralspindlin complex, nucleus, spindle, gamma-tubulin complex, midbody, cytosol, kinetochore, spindle midzone, nuclear condensin complex, cleavage furrow, nucleoplasm, microtubule organizing center |
| P36 | DEG_Nend_UBRbox_1 | MKFH | 1-4 | N-terminal motif that initiates protein degradation by binding to the UBR-box of N-recognins. This N-degron variant comprises N-terminal Arg or Lys as destabilizing residue | cytosol |
|  | DOC_MAPK_MEF2A_6 | KSLVFLGI | 2-9 | A kinase docking motif that mediates interaction towards the ERK1/2 and p38 subfamilies of MAP kinases | nucleus, cytosol, Transcription factor complex |
|  | LIG_BRCT_BRCA1_1 | KSLVF | 2-6 | Phosphopeptide motif which directly interacts with the BRCT (carboxy-terminal) domain of the Breast Cancer Gene BRCA1 with low affinity | nucleus, BRCA1-BARD1 complex |
|  | LIG_LIR_Nem_3 | SLVFLGI | 3-9 | Nematode-specific variant of the canonical LIR motif that binds to Atg8 protein family members to mediate processes involved in autophagy. | cytosol,  cytoplasmic side of late endosome membrane |
|  | MOD_Plk_1 | QECSLQS | 18-24 | Ser/Thr residue phosphorylated by the Plk1 kinase | Central spindlin complex, nucleus,  spindle, gamma-tubulin complex,  midbody, cytosol, kinetochore,  spindle midzone, nuclear condensin complex, cleavage furrow, nucleoplasm, microtubule organizing center |
| P39 | DEG_Nend_UBRbox_1 | MKFL | 1-4 | N-terminal motif that initiates protein degradation by binding to the UBR-box of N-recognins. This N-degron variant comprises N-terminal Arg or Lys as destabilizing residue | cytosol |
|  | DOC_MAPK_MEF2A_6 | KFLVFLGI | 2-9 | A kinase docking motif that mediates interaction towards the ERK1/2 and p38 subfamilies of MAP kinases | nucleus, cytosol, Transcription factor complex |
|  | LIG_14-3-3_CterR_2 | RSCT | 23-26 | C-terminal Arg-containing phospho-motif mediating a strong interaction with 14-3-3 proteins. | nucleus, internal side of plasma membrane, cytosol |
|  | MOD_OFUCOSY | CSLRSC | 20-25 | Site for attachment of a fucose residue to a serine | extracellular |
|  | MOD_Plk_1 | QECSLRS | 18-24 | Ser/Thr residue phosphorylated by the Plk1 kinase | centralspindlin complex, nucleus,  spindle, gamma-tubulin complex,  midbody, cytosol, kinetochore,  spindle midzone, nuclear condensin complex, cleavage furrow, nucleoplasm, microtubule organizing center |
| P42 | DEG_Nend_UBRbox_1 | MKFL | 1-4 | N-terminal motif that initiates protein degradation by binding to the UBR-box of N-recognins. This N-degron variant comprises N-terminal Arg or Lys as destabilizing residue | cytosol |
|  | DOC_MAPK_MEF2A_6 | KFLVFLGI | 2-9 | A kinase docking motif that mediates interaction towards the ERK1/2 and p38 subfamilies of MAP kinases | nucleus, cytosol, Transcription factor complex |
|  | MOD_GSK3_1 | GIITTVAT | 8-15 | GSK3 phosphorylation recognition site | cytosol, nucleus |
|  | MOD_OFUCOSY | CSLQSC | 20-25 | Site for attachment of a fucose residue to a serine | extracellular |
|  | MOD_Plk_1 | QECSLQS | 18-24 | Ser/Thr residue phosphorylated by the Plk1 kinase | centralspindlin complex, nucleus,  spindle, gamma-tubulin complex,  midbody, cytosol, kinetochore,  spindle midzone, nuclear condensin complex, cleavage furrow, nucleoplasm, microtubule organizing center |
| P43 | DEG_Nend_UBRbox_1 | MKFL | 1-4 | N-terminal motif that initiates protein degradation by binding to the UBR-box of N-recognins. This N-degron variant comprises N-terminal Arg or Lys as destabilizing residue | cytosol |
|  | DOC_MAPK_MEF2A_6 | KFLVFLGI | 2-9 | A kinase docking motif that mediates interaction towards the ERK1/2 and p38 subfamilies of MAP kinases | nucleus, cytosol, Transcription factor complex |
|  | LIG_FHA_1 | IITTVVA | 9-15 | Phosphothreonine motif binding a subset of FHA domains that show a preference for a large aliphatic amino acid at the pT+3 position. | nucleus |
|  | MOD_OFUCOSY | CSLQSC | 20-25 | Site for attachment of a fucose residue to a serine | extracellular |
|  | MOD_Plk_1 | QECSLQS | 18-24 | Ser/Thr residue phosphorylated by the Plk1 kinase | centralspindlin complex, nucleus,  spindle, gamma-tubulin complex,  midbody, cytosol, kinetochore,  spindle midzone, nuclear condensin complex, cleavage furrow, nucleoplasm, microtubule organizing center |
|  | MOD_Plk_4 | IITTVVA | 9-15 | Ser/Thr residue phosphorylated by Plk4 | nucleus, cytosol, SCF ubiquitin ligase complex,  cleavage furrow, centriole,  gamma-tubulin ring complex, centriolar satellite, pericentriolar material |
| P45 | DEG_Nend_UBRbox_1 | MKFL | 1-4 | N-terminal motif that initiates protein degradation by binding to the UBR-box of N-recognins. This N-degron variant comprises N-terminal Arg or Lys as destabilizing residue | cytosol |
|  | DOC_MAPK_MEF2A_6 | KFLVFLVI | 2-9 | A kinase docking motif that mediates interaction towards the ERK1/2 and p38 subfamilies of MAP kinases | nucleus, cytosol, Transcription factor complex |
|  | LIG_SUMO_SIM_par_1 | FLVIITT | 6-12 | Motif for the parallel beta augmentation mode of non-covalent binding to SUMO protein. | PML body, nucleus, nuclear body |
|  | MOD_OFUCOSY | CSLQSC | 20-25 | Site for attachment of a fucose residue to a serine | extracellular |
|  | MOD_Plk_1 | QECSLQS | 18-24 | Ser/Thr residue phosphorylated by the Plk1 kinase | centralspindlin complex, nucleus,  spindle, gamma-tubulin complex,  midbody, cytosol, kinetochore,  spindle midzone, nuclear condensin complex, cleavage furrow, nucleoplasm, microtubule organizing center |

**Supplementary Table S4.** Unique polar/non-polar sequence variants of T-ORF8 and their frequencies

| **Serial name** | **The unique polar/non-polar sequence of T-ORF8** | **Frequency of identical sequence** |
| --- | --- | --- |
| P1 | PQPQPPPPPPQQPPPPQQQQQPQQQQ | 1 |
| P12 | PQPPPPPPPPQQPPPPQQQQQPQPQQ | 1 |
| P13 | PQPPPPPPPPQQPPPPQQQQQPQQQP | 1 |
| P16 | PQPPPPPPPPQQPPPPQQQQQPQQPQ | 1 |
| P21 | PQPPPPPPPPQQPPPPQQPQQPQQQQ | 1 |
| P25 | PQPPPPPPPPQQPPPQQQQQQPQQQQ | 1 |
| P28 | PQPPPPPPPPQQPQPPQQQQQPQQQQ | 2 |
| P30 | PQPPPPPPPPXQPPPPQQQQQPQQQQ | 1 |
| P31 | PQPPPPPPPQQQPPPPQQQQQPQQQQ | 1 |
| P32 | PQPPPPPPQPQQPPPPQQQQQPQQQQ | 1 |
| P36 | PQQPPPPPPPQQPPPPQQQQQPQQQQ | 1 |
| P4 | PQPPPPPQPPQQPPPPQQQQQPQQQQ | 2 |
| P40 | PQPPPPPPPPQQPPPPQQXQQPQQQQ | 1 |
| P42 | PQPPPPPPPPQQPPQPQQQQQPQQQQ | 1 |
| **P15** | **PQPPPPPPPPQQPPPPQQQQQPQQQQ** | **28** |
| P5 | PQPPPPPPPPPQPPPPQQQQQPQQQQ | 2 |
| P8 | PQPPPPPPPPQPPPPPQQQQQPQQQQ | 1 |

**Supplementary Table S5.** Frequency of amino acids present in the 47 unique T-ORF8 variants. (Standard single-letter amino acid codes were used.)

|  | **A** | **R** | **N** | **D** | **C** | **Q** | **E** | **G** | **H** | **I** | **L** | **K** | **M** | **F** | **P** | **S** | **T** | **W** | **Y** | **V** |
| --- | --- | --- | --- | --- | --- | --- | --- | --- | --- | --- | --- | --- | --- | --- | --- | --- | --- | --- | --- | --- |
| P47 | 2 | 0 | 0 | 0 | 2 | 2 | 1 | 1 | 1 | 2 | 2 | 1 | 1 | 4 | 0 | 2 | 3 | 0 | 0 | 2 |
| P1 | 2 | 0 | 0 | 0 | 2 | 2 | 1 | 1 | 2 | 2 | 2 | 1 | 1 | 3 | 0 | 2 | 3 | 0 | 0 | 2 |
| P2 | 2 | 0 | 0 | 0 | 2 | 2 | 1 | 1 | 1 | 3 | 3 | 1 | 1 | 3 | 0 | 2 | 3 | 0 | 0 | 1 |
| P3 | 2 | 0 | 0 | 0 | 2 | 2 | 1 | 1 | 1 | 2 | 2 | 1 | 1 | 4 | 0 | 2 | 3 | 0 | 0 | 2 |
| P4 | 2 | 0 | 0 | 0 | 2 | 2 | 2 | 0 | 1 | 2 | 3 | 1 | 1 | 3 | 0 | 2 | 3 | 0 | 0 | 2 |
| P5 | 3 | 0 | 0 | 0 | 2 | 2 | 1 | 1 | 1 | 2 | 3 | 1 | 1 | 3 | 0 | 2 | 2 | 0 | 0 | 2 |
| P6 | 2 | 0 | 0 | 0 | 2 | 2 | 1 | 1 | 1 | 3 | 3 | 1 | 1 | 3 | 0 | 2 | 2 | 0 | 0 | 2 |
| P7 | 2 | 0 | 0 | 0 | 2 | 2 | 1 | 1 | 1 | 2 | 3 | 2 | 1 | 3 | 0 | 2 | 2 | 0 | 0 | 2 |
| P8 | 2 | 0 | 0 | 0 | 2 | 2 | 1 | 1 | 1 | 2 | 3 | 1 | 1 | 3 | 1 | 2 | 2 | 0 | 0 | 2 |
| P37 | 3 | 0 | 0 | 0 | 2 | 2 | 1 | 1 | 1 | 2 | 3 | 1 | 1 | 3 | 0 | 2 | 3 | 0 | 0 | 1 |
| P9 | 2 | 0 | 0 | 0 | 2 | 2 | 1 | 1 | 1 | 3 | 3 | 1 | 1 | 3 | 0 | 2 | 3 | 0 | 0 | 1 |
| P10 | 2 | 0 | 0 | 0 | 2 | 2 | 1 | 1 | 1 | 2 | 4 | 1 | 1 | 3 | 0 | 2 | 3 | 0 | 0 | 1 |
| P38 | 2 | 0 | 0 | 1 | 2 | 2 | 1 | 1 | 0 | 2 | 3 | 1 | 1 | 3 | 0 | 2 | 3 | 0 | 0 | 2 |
| P11 | 2 | 0 | 0 | 1 | 2 | 2 | 0 | 1 | 1 | 2 | 3 | 1 | 1 | 3 | 0 | 2 | 3 | 0 | 0 | 2 |
| P12 | 2 | 0 | 0 | 0 | 2 | 2 | 1 | 1 | 1 | 2 | 4 | 1 | 1 | 3 | 0 | 1 | 3 | 0 | 0 | 2 |
| P13 | 2 | 0 | 0 | 0 | 2 | 2 | 1 | 1 | 1 | 3 | 3 | 1 | 1 | 3 | 0 | 2 | 2 | 0 | 0 | 2 |
| P14 | 2 | 0 | 1 | 0 | 2 | 2 | 1 | 1 | 1 | 2 | 3 | 1 | 1 | 3 | 0 | 2 | 2 | 0 | 0 | 2 |
| P15 | 2 | 0 | 0 | 0 | 2 | 2 | 1 | 1 | 1 | 2 | 3 | 1 | 1 | 3 | 0 | 2 | 3 | 0 | 0 | 2 |
| P16 | 2 | 0 | 0 | 0 | 1 | 2 | 1 | 1 | 1 | 2 | 3 | 1 | 1 | 4 | 0 | 2 | 3 | 0 | 0 | 2 |
| P17 | 2 | 1 | 0 | 0 | 1 | 2 | 1 | 1 | 1 | 2 | 3 | 1 | 1 | 3 | 0 | 2 | 3 | 0 | 0 | 2 |
| P18 | 2 | 0 | 0 | 0 | 1 | 2 | 1 | 1 | 1 | 2 | 3 | 1 | 1 | 3 | 0 | 3 | 3 | 0 | 0 | 2 |
| P39 | 2 | 1 | 0 | 0 | 2 | 1 | 1 | 1 | 1 | 2 | 3 | 1 | 1 | 3 | 0 | 2 | 3 | 0 | 0 | 2 |
| P19 | 2 | 1 | 0 | 0 | 1 | 2 | 1 | 1 | 1 | 2 | 3 | 1 | 1 | 3 | 0 | 2 | 3 | 0 | 0 | 2 |
| P20 | 2 | 0 | 0 | 0 | 1 | 2 | 1 | 1 | 1 | 2 | 3 | 1 | 1 | 3 | 0 | 2 | 3 | 0 | 1 | 2 |
| P21 | 2 | 0 | 0 | 0 | 2 | 2 | 0 | 2 | 1 | 2 | 3 | 1 | 1 | 3 | 0 | 2 | 3 | 0 | 0 | 2 |
| P40 | 2 | 0 | 0 | 0 | 2 | 2 | 0 | 1 | 1 | 2 | 3 | 1 | 1 | 3 | 0 | 2 | 3 | 0 | 0 | 2 |
| P41 | 2 | 0 | 1 | 0 | 2 | 2 | 1 | 1 | 0 | 2 | 3 | 1 | 1 | 3 | 0 | 2 | 3 | 0 | 0 | 2 |
| P22 | 2 | 1 | 0 | 0 | 2 | 2 | 1 | 1 | 0 | 2 | 3 | 1 | 1 | 3 | 0 | 2 | 3 | 0 | 0 | 2 |
| P23 | 2 | 0 | 0 | 0 | 2 | 2 | 1 | 1 | 0 | 2 | 3 | 1 | 1 | 3 | 0 | 2 | 3 | 0 | 1 | 2 |
| P24 | 2 | 0 | 0 | 0 | 2 | 2 | 1 | 1 | 1 | 2 | 4 | 1 | 1 | 2 | 0 | 2 | 3 | 0 | 0 | 2 |
| P25 | 2 | 0 | 0 | 0 | 2 | 2 | 1 | 1 | 1 | 2 | 3 | 1 | 1 | 2 | 0 | 3 | 3 | 0 | 0 | 2 |
| P26 | 2 | 0 | 0 | 0 | 2 | 2 | 1 | 1 | 1 | 2 | 3 | 1 | 1 | 2 | 0 | 2 | 3 | 0 | 0 | 3 |
| P42 | 1 | 0 | 0 | 0 | 2 | 2 | 1 | 1 | 1 | 2 | 3 | 1 | 1 | 3 | 0 | 2 | 4 | 0 | 0 | 2 |
| P27 | 1 | 0 | 0 | 0 | 2 | 2 | 1 | 1 | 1 | 2 | 3 | 1 | 1 | 3 | 0 | 2 | 3 | 0 | 0 | 3 |
| P28 | 1 | 0 | 0 | 0 | 2 | 2 | 1 | 1 | 1 | 2 | 3 | 1 | 1 | 3 | 0 | 3 | 3 | 0 | 0 | 2 |
| P29 | 1 | 0 | 0 | 0 | 2 | 2 | 1 | 1 | 1 | 2 | 3 | 1 | 1 | 3 | 0 | 2 | 4 | 0 | 0 | 2 |
| P43 | 1 | 0 | 0 | 0 | 2 | 2 | 1 | 1 | 1 | 2 | 3 | 1 | 1 | 3 | 0 | 2 | 3 | 0 | 0 | 3 |
| P30 | 2 | 0 | 0 | 0 | 2 | 2 | 1 | 1 | 1 | 2 | 3 | 1 | 1 | 3 | 0 | 2 | 2 | 0 | 0 | 2 |
| P44 | 2 | 0 | 0 | 0 | 2 | 2 | 1 | 1 | 1 | 1 | 3 | 1 | 2 | 3 | 0 | 2 | 3 | 0 | 0 | 2 |
| P31 | 2 | 0 | 0 | 0 | 2 | 2 | 1 | 1 | 1 | 1 | 3 | 1 | 1 | 3 | 0 | 2 | 4 | 0 | 0 | 2 |
| P32 | 2 | 0 | 0 | 0 | 2 | 2 | 1 | 1 | 1 | 1 | 3 | 1 | 1 | 3 | 0 | 2 | 4 | 0 | 0 | 2 |
| P33 | 2 | 1 | 0 | 0 | 2 | 2 | 1 | 0 | 1 | 2 | 3 | 1 | 1 | 3 | 0 | 2 | 3 | 0 | 0 | 2 |
| P45 | 2 | 0 | 0 | 0 | 2 | 2 | 1 | 0 | 1 | 2 | 3 | 1 | 1 | 3 | 0 | 2 | 3 | 0 | 0 | 3 |
| P34 | 2 | 0 | 0 | 0 | 2 | 2 | 1 | 1 | 1 | 2 | 4 | 1 | 1 | 2 | 0 | 2 | 3 | 0 | 0 | 2 |
| P46 | 2 | 0 | 0 | 0 | 2 | 2 | 1 | 1 | 1 | 3 | 3 | 1 | 1 | 2 | 0 | 2 | 3 | 0 | 0 | 2 |
| P35 | 2 | 0 | 0 | 0 | 2 | 2 | 1 | 1 | 1 | 2 | 4 | 1 | 1 | 2 | 0 | 2 | 3 | 0 | 0 | 2 |
| P36 | 2 | 0 | 0 | 0 | 2 | 2 | 1 | 1 | 1 | 2 | 3 | 1 | 1 | 2 | 0 | 3 | 3 | 0 | 0 | 2 |

**Supplementary Table S6**. Euclidean distances calculated for each pair of frequency vectors corresponding to all unique T-ORF8 variants

|  | P47 | P1 | P2 | P3 | P4 | P5 | P6 | P7 | P8 | P37 | P9 | P10 | P38 | P11 | P12 | P13 | P14 | **P15** | P16 | P17 | P18 | P39 | P19 | P20 | P21 | P40 | P41 | P22 | P23 | P24 | P25 | P26 | P42 | P27 | P28 | P29 | P43 | P30 | P44 | P31 | P32 | P33 | P45 | P34 | P46 | P35 | P36 |
| --- | --- | --- | --- | --- | --- | --- | --- | --- | --- | --- | --- | --- | --- | --- | --- | --- | --- | --- | --- | --- | --- | --- | --- | --- | --- | --- | --- | --- | --- | --- | --- | --- | --- | --- | --- | --- | --- | --- | --- | --- | --- | --- | --- | --- | --- | --- | --- |
| P47 | 0.00 | 1.41 | 2.00 | 0.00 | 2.00 | 2.00 | 2.00 | 2.00 | 2.00 | 2.00 | 2.00 | 2.45 | 2.00 | 2.00 | 2.45 | 2.00 | 2.00 | **1.41** | 1.41 | 2.00 | 2.00 | 2.00 | 2.00 | 2.00 | 2.00 | 1.73 | 2.00 | 2.00 | 2.00 | 2.83 | 2.45 | 2.45 | 2.00 | 2.00 | 2.00 | 2.00 | 2.00 | 1.73 | 2.00 | 2.00 | 2.00 | 2.00 | 2.00 | 2.83 | 2.45 | 2.83 | 2.45 |
| P1 | 1.41 | 0.00 | 2.00 | 1.41 | 2.00 | 2.00 | 2.00 | 2.00 | 2.00 | 2.00 | 2.00 | 2.45 | 2.45 | 2.00 | 2.45 | 2.00 | 2.00 | **1.41** | 2.00 | 2.00 | 2.00 | 2.00 | 2.00 | 2.00 | 2.00 | 1.73 | 2.45 | 2.45 | 2.45 | 2.45 | 2.00 | 2.00 | 2.00 | 2.00 | 2.00 | 2.00 | 2.00 | 1.73 | 2.00 | 2.00 | 2.00 | 2.00 | 2.00 | 2.45 | 2.00 | 2.45 | 2.00 |
| P2 | 2.00 | 2.00 | 0.00 | 2.00 | 2.00 | 2.00 | 1.41 | 2.00 | 2.00 | 1.41 | 0.00 | 1.41 | 2.00 | 2.00 | 2.00 | 1.41 | 2.00 | **1.41** | 2.00 | 2.00 | 2.00 | 2.00 | 2.00 | 2.00 | 2.00 | 1.73 | 2.00 | 2.00 | 2.00 | 2.00 | 2.00 | 2.45 | 2.00 | 2.45 | 2.00 | 2.00 | 2.45 | 1.73 | 2.45 | 2.45 | 2.45 | 2.00 | 2.45 | 2.00 | 1.41 | 2.00 | 2.00 |
| P3 | 0.00 | 1.41 | 2.00 | 0.00 | 2.00 | 2.00 | 2.00 | 2.00 | 2.00 | 2.00 | 2.00 | 2.45 | 2.00 | 2.00 | 2.45 | 2.00 | 2.00 | **1.41** | 1.41 | 2.00 | 2.00 | 2.00 | 2.00 | 2.00 | 2.00 | 1.73 | 2.00 | 2.00 | 2.00 | 2.83 | 2.45 | 2.45 | 2.00 | 2.00 | 2.00 | 2.00 | 2.00 | 1.73 | 2.00 | 2.00 | 2.00 | 2.00 | 2.00 | 2.83 | 2.45 | 2.83 | 2.45 |
| P4 | 2.00 | 2.00 | 2.00 | 2.00 | 0.00 | 2.00 | 2.00 | 2.00 | 2.00 | 2.00 | 2.00 | 2.00 | 2.00 | 2.45 | 2.00 | 2.00 | 2.00 | **1.41** | 2.00 | 2.00 | 2.00 | 2.00 | 2.00 | 2.00 | 2.83 | 2.24 | 2.00 | 2.00 | 2.00 | 2.00 | 2.00 | 2.00 | 2.00 | 2.00 | 2.00 | 2.00 | 2.00 | 1.73 | 2.00 | 2.00 | 2.00 | 1.41 | 1.41 | 2.00 | 2.00 | 2.00 | 2.00 |
| P5 | 2.00 | 2.00 | 2.00 | 2.00 | 2.00 | 0.00 | 1.41 | 1.41 | 1.41 | 1.41 | 2.00 | 2.00 | 2.00 | 2.00 | 2.00 | 1.41 | 1.41 | **1.41** | 2.00 | 2.00 | 2.00 | 2.00 | 2.00 | 2.00 | 2.00 | 1.73 | 2.00 | 2.00 | 2.00 | 2.00 | 2.00 | 2.00 | 2.83 | 2.45 | 2.45 | 2.83 | 2.45 | 1.00 | 2.00 | 2.45 | 2.45 | 2.00 | 2.00 | 2.00 | 2.00 | 2.00 | 2.00 |
| P6 | 2.00 | 2.00 | 1.41 | 2.00 | 2.00 | 1.41 | 0.00 | 1.41 | 1.41 | 2.00 | 1.41 | 2.00 | 2.00 | 2.00 | 2.00 | 0.00 | 1.41 | **1.41** | 2.00 | 2.00 | 2.00 | 2.00 | 2.00 | 2.00 | 2.00 | 1.73 | 2.00 | 2.00 | 2.00 | 2.00 | 2.00 | 2.00 | 2.45 | 2.00 | 2.00 | 2.45 | 2.00 | 1.00 | 2.45 | 2.83 | 2.83 | 2.00 | 2.00 | 2.00 | 1.41 | 2.00 | 2.00 |
| P7 | 2.00 | 2.00 | 2.00 | 2.00 | 2.00 | 1.41 | 1.41 | 0.00 | 1.41 | 2.00 | 2.00 | 2.00 | 2.00 | 2.00 | 2.00 | 1.41 | 1.41 | **1.41** | 2.00 | 2.00 | 2.00 | 2.00 | 2.00 | 2.00 | 2.00 | 1.73 | 2.00 | 2.00 | 2.00 | 2.00 | 2.00 | 2.00 | 2.45 | 2.00 | 2.00 | 2.45 | 2.00 | 1.00 | 2.00 | 2.45 | 2.45 | 2.00 | 2.00 | 2.00 | 2.00 | 2.00 | 2.00 |
| P8 | 2.00 | 2.00 | 2.00 | 2.00 | 2.00 | 1.41 | 1.41 | 1.41 | 0.00 | 2.00 | 2.00 | 2.00 | 2.00 | 2.00 | 2.00 | 1.41 | 1.41 | **1.41** | 2.00 | 2.00 | 2.00 | 2.00 | 2.00 | 2.00 | 2.00 | 1.73 | 2.00 | 2.00 | 2.00 | 2.00 | 2.00 | 2.00 | 2.45 | 2.00 | 2.00 | 2.45 | 2.00 | 1.00 | 2.00 | 2.45 | 2.45 | 2.00 | 2.00 | 2.00 | 2.00 | 2.00 | 2.00 |
| P37 | 2.00 | 2.00 | 1.41 | 2.00 | 2.00 | 1.41 | 2.00 | 2.00 | 2.00 | 0.00 | 1.41 | 1.41 | 2.00 | 2.00 | 2.00 | 2.00 | 2.00 | **1.41** | 2.00 | 2.00 | 2.00 | 2.00 | 2.00 | 2.00 | 2.00 | 1.73 | 2.00 | 2.00 | 2.00 | 2.00 | 2.00 | 2.45 | 2.45 | 2.83 | 2.45 | 2.45 | 2.83 | 1.73 | 2.00 | 2.00 | 2.00 | 2.00 | 2.45 | 2.00 | 2.00 | 2.00 | 2.00 |
| P9 | 2.00 | 2.00 | 0.00 | 2.00 | 2.00 | 2.00 | 1.41 | 2.00 | 2.00 | 1.41 | 0.00 | 1.41 | 2.00 | 2.00 | 2.00 | 1.41 | 2.00 | **1.41** | 2.00 | 2.00 | 2.00 | 2.00 | 2.00 | 2.00 | 2.00 | 1.73 | 2.00 | 2.00 | 2.00 | 2.00 | 2.00 | 2.45 | 2.00 | 2.45 | 2.00 | 2.00 | 2.45 | 1.73 | 2.45 | 2.45 | 2.45 | 2.00 | 2.45 | 2.00 | 1.41 | 2.00 | 2.00 |
| P10 | 2.45 | 2.45 | 1.41 | 2.45 | 2.00 | 2.00 | 2.00 | 2.00 | 2.00 | 1.41 | 1.41 | 0.00 | 2.00 | 2.00 | 1.41 | 2.00 | 2.00 | **1.41** | 2.00 | 2.00 | 2.00 | 2.00 | 2.00 | 2.00 | 2.00 | 1.73 | 2.00 | 2.00 | 2.00 | 1.41 | 2.00 | 2.45 | 2.00 | 2.45 | 2.00 | 2.00 | 2.45 | 1.73 | 2.00 | 2.00 | 2.00 | 2.00 | 2.45 | 1.41 | 2.00 | 1.41 | 2.00 |
| P38 | 2.00 | 2.45 | 2.00 | 2.00 | 2.00 | 2.00 | 2.00 | 2.00 | 2.00 | 2.00 | 2.00 | 2.00 | 0.00 | 1.41 | 2.00 | 2.00 | 2.00 | **1.41** | 2.00 | 2.00 | 2.00 | 2.00 | 2.00 | 2.00 | 2.00 | 1.73 | 1.41 | 1.41 | 1.41 | 2.00 | 2.00 | 2.00 | 2.00 | 2.00 | 2.00 | 2.00 | 2.00 | 1.73 | 2.00 | 2.00 | 2.00 | 2.00 | 2.00 | 2.00 | 2.00 | 2.00 | 2.00 |
| P11 | 2.00 | 2.00 | 2.00 | 2.00 | 2.45 | 2.00 | 2.00 | 2.00 | 2.00 | 2.00 | 2.00 | 2.00 | 1.41 | 0.00 | 2.00 | 2.00 | 2.00 | **1.41** | 2.00 | 2.00 | 2.00 | 2.00 | 2.00 | 2.00 | 1.41 | 1.00 | 2.00 | 2.00 | 2.00 | 2.00 | 2.00 | 2.00 | 2.00 | 2.00 | 2.00 | 2.00 | 2.00 | 1.73 | 2.00 | 2.00 | 2.00 | 2.00 | 2.00 | 2.00 | 2.00 | 2.00 | 2.00 |
| P12 | 2.45 | 2.45 | 2.00 | 2.45 | 2.00 | 2.00 | 2.00 | 2.00 | 2.00 | 2.00 | 2.00 | 1.41 | 2.00 | 2.00 | 0.00 | 2.00 | 2.00 | **1.41** | 2.00 | 2.00 | 2.45 | 2.00 | 2.00 | 2.00 | 2.00 | 1.73 | 2.00 | 2.00 | 2.00 | 1.41 | 2.45 | 2.00 | 2.00 | 2.00 | 2.45 | 2.00 | 2.00 | 1.73 | 2.00 | 2.00 | 2.00 | 2.00 | 2.00 | 1.41 | 2.00 | 1.41 | 2.45 |
| P13 | 2.00 | 2.00 | 1.41 | 2.00 | 2.00 | 1.41 | 0.00 | 1.41 | 1.41 | 2.00 | 1.41 | 2.00 | 2.00 | 2.00 | 2.00 | 0.00 | 1.41 | **1.41** | 2.00 | 2.00 | 2.00 | 2.00 | 2.00 | 2.00 | 2.00 | 1.73 | 2.00 | 2.00 | 2.00 | 2.00 | 2.00 | 2.00 | 2.45 | 2.00 | 2.00 | 2.45 | 2.00 | 1.00 | 2.45 | 2.83 | 2.83 | 2.00 | 2.00 | 2.00 | 1.41 | 2.00 | 2.00 |
| P14 | 2.00 | 2.00 | 2.00 | 2.00 | 2.00 | 1.41 | 1.41 | 1.41 | 1.41 | 2.00 | 2.00 | 2.00 | 2.00 | 2.00 | 2.00 | 1.41 | 0.00 | **1.41** | 2.00 | 2.00 | 2.00 | 2.00 | 2.00 | 2.00 | 2.00 | 1.73 | 1.41 | 2.00 | 2.00 | 2.00 | 2.00 | 2.00 | 2.45 | 2.00 | 2.00 | 2.45 | 2.00 | 1.00 | 2.00 | 2.45 | 2.45 | 2.00 | 2.00 | 2.00 | 2.00 | 2.00 | 2.00 |
| P15 | **1.41** | **1.41** | **1.41** | **1.41** | **1.41** | **1.41** | **1.41** | **1.41** | **1.41** | **1.41** | **1.41** | **1.41** | **1.41** | **1.41** | **1.41** | **1.41** | **1.41** | **0.00** | **1.41** | **1.41** | **1.41** | **1.41** | **1.41** | **1.41** | **1.41** | **1.00** | **1.41** | **1.41** | **1.41** | **1.41** | **1.41** | **1.41** | **1.41** | **1.41** | **1.41** | **1.41** | **1.41** | **1.00** | **1.41** | **1.41** | **1.41** | **1.41** | **1.41** | **1.41** | **1.41** | **1.41** | **1.41** |
| P16 | 1.41 | 2.00 | 2.00 | 1.41 | 2.00 | 2.00 | 2.00 | 2.00 | 2.00 | 2.00 | 2.00 | 2.00 | 2.00 | 2.00 | 2.00 | 2.00 | 2.00 | **1.41** | 0.00 | 1.41 | 1.41 | 2.00 | 1.41 | 1.41 | 2.00 | 1.73 | 2.00 | 2.00 | 2.00 | 2.45 | 2.45 | 2.45 | 2.00 | 2.00 | 2.00 | 2.00 | 2.00 | 1.73 | 2.00 | 2.00 | 2.00 | 2.00 | 2.00 | 2.45 | 2.45 | 2.45 | 2.45 |
| P17 | 2.00 | 2.00 | 2.00 | 2.00 | 2.00 | 2.00 | 2.00 | 2.00 | 2.00 | 2.00 | 2.00 | 2.00 | 2.00 | 2.00 | 2.00 | 2.00 | 2.00 | **1.41** | 1.41 | 0.00 | 1.41 | 1.41 | 0.00 | 1.41 | 2.00 | 1.73 | 2.00 | 1.41 | 2.00 | 2.00 | 2.00 | 2.00 | 2.00 | 2.00 | 2.00 | 2.00 | 2.00 | 1.73 | 2.00 | 2.00 | 2.00 | 1.41 | 2.00 | 2.00 | 2.00 | 2.00 | 2.00 |
| P18 | 2.00 | 2.00 | 2.00 | 2.00 | 2.00 | 2.00 | 2.00 | 2.00 | 2.00 | 2.00 | 2.00 | 2.00 | 2.00 | 2.00 | 2.45 | 2.00 | 2.00 | **1.41** | 1.41 | 1.41 | 0.00 | 2.00 | 1.41 | 1.41 | 2.00 | 1.73 | 2.00 | 2.00 | 2.00 | 2.00 | 1.41 | 2.00 | 2.00 | 2.00 | 1.41 | 2.00 | 2.00 | 1.73 | 2.00 | 2.00 | 2.00 | 2.00 | 2.00 | 2.00 | 2.00 | 2.00 | 1.41 |
| P39 | 2.00 | 2.00 | 2.00 | 2.00 | 2.00 | 2.00 | 2.00 | 2.00 | 2.00 | 2.00 | 2.00 | 2.00 | 2.00 | 2.00 | 2.00 | 2.00 | 2.00 | **1.41** | 2.00 | 1.41 | 2.00 | 0.00 | 1.41 | 2.00 | 2.00 | 1.73 | 2.00 | 1.41 | 2.00 | 2.00 | 2.00 | 2.00 | 2.00 | 2.00 | 2.00 | 2.00 | 2.00 | 1.73 | 2.00 | 2.00 | 2.00 | 1.41 | 2.00 | 2.00 | 2.00 | 2.00 | 2.00 |
| P19 | 2.00 | 2.00 | 2.00 | 2.00 | 2.00 | 2.00 | 2.00 | 2.00 | 2.00 | 2.00 | 2.00 | 2.00 | 2.00 | 2.00 | 2.00 | 2.00 | 2.00 | **1.41** | 1.41 | 0.00 | 1.41 | 1.41 | 0.00 | 1.41 | 2.00 | 1.73 | 2.00 | 1.41 | 2.00 | 2.00 | 2.00 | 2.00 | 2.00 | 2.00 | 2.00 | 2.00 | 2.00 | 1.73 | 2.00 | 2.00 | 2.00 | 1.41 | 2.00 | 2.00 | 2.00 | 2.00 | 2.00 |
| P20 | 2.00 | 2.00 | 2.00 | 2.00 | 2.00 | 2.00 | 2.00 | 2.00 | 2.00 | 2.00 | 2.00 | 2.00 | 2.00 | 2.00 | 2.00 | 2.00 | 2.00 | **1.41** | 1.41 | 1.41 | 1.41 | 2.00 | 1.41 | 0.00 | 2.00 | 1.73 | 2.00 | 2.00 | 1.41 | 2.00 | 2.00 | 2.00 | 2.00 | 2.00 | 2.00 | 2.00 | 2.00 | 1.73 | 2.00 | 2.00 | 2.00 | 2.00 | 2.00 | 2.00 | 2.00 | 2.00 | 2.00 |
| P21 | 2.00 | 2.00 | 2.00 | 2.00 | 2.83 | 2.00 | 2.00 | 2.00 | 2.00 | 2.00 | 2.00 | 2.00 | 2.00 | 1.41 | 2.00 | 2.00 | 2.00 | **1.41** | 2.00 | 2.00 | 2.00 | 2.00 | 2.00 | 2.00 | 0.00 | 1.00 | 2.00 | 2.00 | 2.00 | 2.00 | 2.00 | 2.00 | 2.00 | 2.00 | 2.00 | 2.00 | 2.00 | 1.73 | 2.00 | 2.00 | 2.00 | 2.45 | 2.45 | 2.00 | 2.00 | 2.00 | 2.00 |
| P40 | 1.73 | 1.73 | 1.73 | 1.73 | 2.24 | 1.73 | 1.73 | 1.73 | 1.73 | 1.73 | 1.73 | 1.73 | 1.73 | 1.00 | 1.73 | 1.73 | 1.73 | **1.00** | 1.73 | 1.73 | 1.73 | 1.73 | 1.73 | 1.73 | 1.00 | 0.00 | 1.73 | 1.73 | 1.73 | 1.73 | 1.73 | 1.73 | 1.73 | 1.73 | 1.73 | 1.73 | 1.73 | 1.41 | 1.73 | 1.73 | 1.73 | 1.73 | 1.73 | 1.73 | 1.73 | 1.73 | 1.73 |
| P41 | 2.00 | 2.45 | 2.00 | 2.00 | 2.00 | 2.00 | 2.00 | 2.00 | 2.00 | 2.00 | 2.00 | 2.00 | 1.41 | 2.00 | 2.00 | 2.00 | 1.41 | **1.41** | 2.00 | 2.00 | 2.00 | 2.00 | 2.00 | 2.00 | 2.00 | 1.73 | 0.00 | 1.41 | 1.41 | 2.00 | 2.00 | 2.00 | 2.00 | 2.00 | 2.00 | 2.00 | 2.00 | 1.73 | 2.00 | 2.00 | 2.00 | 2.00 | 2.00 | 2.00 | 2.00 | 2.00 | 2.00 |
| P22 | 2.00 | 2.45 | 2.00 | 2.00 | 2.00 | 2.00 | 2.00 | 2.00 | 2.00 | 2.00 | 2.00 | 2.00 | 1.41 | 2.00 | 2.00 | 2.00 | 2.00 | **1.41** | 2.00 | 1.41 | 2.00 | 1.41 | 1.41 | 2.00 | 2.00 | 1.73 | 1.41 | 0.00 | 1.41 | 2.00 | 2.00 | 2.00 | 2.00 | 2.00 | 2.00 | 2.00 | 2.00 | 1.73 | 2.00 | 2.00 | 2.00 | 1.41 | 2.00 | 2.00 | 2.00 | 2.00 | 2.00 |
| P23 | 2.00 | 2.45 | 2.00 | 2.00 | 2.00 | 2.00 | 2.00 | 2.00 | 2.00 | 2.00 | 2.00 | 2.00 | 1.41 | 2.00 | 2.00 | 2.00 | 2.00 | **1.41** | 2.00 | 2.00 | 2.00 | 2.00 | 2.00 | 1.41 | 2.00 | 1.73 | 1.41 | 1.41 | 0.00 | 2.00 | 2.00 | 2.00 | 2.00 | 2.00 | 2.00 | 2.00 | 2.00 | 1.73 | 2.00 | 2.00 | 2.00 | 2.00 | 2.00 | 2.00 | 2.00 | 2.00 | 2.00 |
| P24 | 2.83 | 2.45 | 2.00 | 2.83 | 2.00 | 2.00 | 2.00 | 2.00 | 2.00 | 2.00 | 2.00 | 1.41 | 2.00 | 2.00 | 1.41 | 2.00 | 2.00 | **1.41** | 2.45 | 2.00 | 2.00 | 2.00 | 2.00 | 2.00 | 2.00 | 1.73 | 2.00 | 2.00 | 2.00 | 0.00 | 1.41 | 1.41 | 2.00 | 2.00 | 2.00 | 2.00 | 2.00 | 1.73 | 2.00 | 2.00 | 2.00 | 2.00 | 2.00 | 0.00 | 1.41 | 0.00 | 1.41 |
| P25 | 2.45 | 2.00 | 2.00 | 2.45 | 2.00 | 2.00 | 2.00 | 2.00 | 2.00 | 2.00 | 2.00 | 2.00 | 2.00 | 2.00 | 2.45 | 2.00 | 2.00 | **1.41** | 2.45 | 2.00 | 1.41 | 2.00 | 2.00 | 2.00 | 2.00 | 1.73 | 2.00 | 2.00 | 2.00 | 1.41 | 0.00 | 1.41 | 2.00 | 2.00 | 1.41 | 2.00 | 2.00 | 1.73 | 2.00 | 2.00 | 2.00 | 2.00 | 2.00 | 1.41 | 1.41 | 1.41 | 0.00 |
| P26 | 2.45 | 2.00 | 2.45 | 2.45 | 2.00 | 2.00 | 2.00 | 2.00 | 2.00 | 2.45 | 2.45 | 2.45 | 2.00 | 2.00 | 2.00 | 2.00 | 2.00 | **1.41** | 2.45 | 2.00 | 2.00 | 2.00 | 2.00 | 2.00 | 2.00 | 1.73 | 2.00 | 2.00 | 2.00 | 1.41 | 1.41 | 0.00 | 2.00 | 1.41 | 2.00 | 2.00 | 1.41 | 1.73 | 2.00 | 2.00 | 2.00 | 2.00 | 1.41 | 1.41 | 1.41 | 1.41 | 1.41 |
| P42 | 2.00 | 2.00 | 2.00 | 2.00 | 2.00 | 2.83 | 2.45 | 2.45 | 2.45 | 2.45 | 2.00 | 2.00 | 2.00 | 2.00 | 2.00 | 2.45 | 2.45 | **1.41** | 2.00 | 2.00 | 2.00 | 2.00 | 2.00 | 2.00 | 2.00 | 1.73 | 2.00 | 2.00 | 2.00 | 2.00 | 2.00 | 2.00 | 0.00 | 1.41 | 1.41 | 0.00 | 1.41 | 2.24 | 2.00 | 1.41 | 1.41 | 2.00 | 2.00 | 2.00 | 2.00 | 2.00 | 2.00 |
| P27 | 2.00 | 2.00 | 2.45 | 2.00 | 2.00 | 2.45 | 2.00 | 2.00 | 2.00 | 2.83 | 2.45 | 2.45 | 2.00 | 2.00 | 2.00 | 2.00 | 2.00 | **1.41** | 2.00 | 2.00 | 2.00 | 2.00 | 2.00 | 2.00 | 2.00 | 1.73 | 2.00 | 2.00 | 2.00 | 2.00 | 2.00 | 1.41 | 1.41 | 0.00 | 1.41 | 1.41 | 0.00 | 1.73 | 2.00 | 2.00 | 2.00 | 2.00 | 1.41 | 2.00 | 2.00 | 2.00 | 2.00 |
| P28 | 2.00 | 2.00 | 2.00 | 2.00 | 2.00 | 2.45 | 2.00 | 2.00 | 2.00 | 2.45 | 2.00 | 2.00 | 2.00 | 2.00 | 2.45 | 2.00 | 2.00 | **1.41** | 2.00 | 2.00 | 1.41 | 2.00 | 2.00 | 2.00 | 2.00 | 1.73 | 2.00 | 2.00 | 2.00 | 2.00 | 1.41 | 2.00 | 1.41 | 1.41 | 0.00 | 1.41 | 1.41 | 1.73 | 2.00 | 2.00 | 2.00 | 2.00 | 2.00 | 2.00 | 2.00 | 2.00 | 1.41 |
| P29 | 2.00 | 2.00 | 2.00 | 2.00 | 2.00 | 2.83 | 2.45 | 2.45 | 2.45 | 2.45 | 2.00 | 2.00 | 2.00 | 2.00 | 2.00 | 2.45 | 2.45 | **1.41** | 2.00 | 2.00 | 2.00 | 2.00 | 2.00 | 2.00 | 2.00 | 1.73 | 2.00 | 2.00 | 2.00 | 2.00 | 2.00 | 2.00 | 0.00 | 1.41 | 1.41 | 0.00 | 1.41 | 2.24 | 2.00 | 1.41 | 1.41 | 2.00 | 2.00 | 2.00 | 2.00 | 2.00 | 2.00 |
| P43 | 2.00 | 2.00 | 2.45 | 2.00 | 2.00 | 2.45 | 2.00 | 2.00 | 2.00 | 2.83 | 2.45 | 2.45 | 2.00 | 2.00 | 2.00 | 2.00 | 2.00 | **1.41** | 2.00 | 2.00 | 2.00 | 2.00 | 2.00 | 2.00 | 2.00 | 1.73 | 2.00 | 2.00 | 2.00 | 2.00 | 2.00 | 1.41 | 1.41 | 0.00 | 1.41 | 1.41 | 0.00 | 1.73 | 2.00 | 2.00 | 2.00 | 2.00 | 1.41 | 2.00 | 2.00 | 2.00 | 2.00 |
| P30 | 1.73 | 1.73 | 1.73 | 1.73 | 1.73 | 1.00 | 1.00 | 1.00 | 1.00 | 1.73 | 1.73 | 1.73 | 1.73 | 1.73 | 1.73 | 1.00 | 1.00 | **1.00** | 1.73 | 1.73 | 1.73 | 1.73 | 1.73 | 1.73 | 1.73 | 1.41 | 1.73 | 1.73 | 1.73 | 1.73 | 1.73 | 1.73 | 2.24 | 1.73 | 1.73 | 2.24 | 1.73 | 0.00 | 1.73 | 2.24 | 2.24 | 1.73 | 1.73 | 1.73 | 1.73 | 1.73 | 1.73 |
| P44 | 2.00 | 2.00 | 2.45 | 2.00 | 2.00 | 2.00 | 2.45 | 2.00 | 2.00 | 2.00 | 2.45 | 2.00 | 2.00 | 2.00 | 2.00 | 2.45 | 2.00 | **1.41** | 2.00 | 2.00 | 2.00 | 2.00 | 2.00 | 2.00 | 2.00 | 1.73 | 2.00 | 2.00 | 2.00 | 2.00 | 2.00 | 2.00 | 2.00 | 2.00 | 2.00 | 2.00 | 2.00 | 1.73 | 0.00 | 1.41 | 1.41 | 2.00 | 2.00 | 2.00 | 2.45 | 2.00 | 2.00 |
| P31 | 2.00 | 2.00 | 2.45 | 2.00 | 2.00 | 2.45 | 2.83 | 2.45 | 2.45 | 2.00 | 2.45 | 2.00 | 2.00 | 2.00 | 2.00 | 2.83 | 2.45 | **1.41** | 2.00 | 2.00 | 2.00 | 2.00 | 2.00 | 2.00 | 2.00 | 1.73 | 2.00 | 2.00 | 2.00 | 2.00 | 2.00 | 2.00 | 1.41 | 2.00 | 2.00 | 1.41 | 2.00 | 2.24 | 1.41 | 0.00 | 0.00 | 2.00 | 2.00 | 2.00 | 2.45 | 2.00 | 2.00 |
| P32 | 2.00 | 2.00 | 2.45 | 2.00 | 2.00 | 2.45 | 2.83 | 2.45 | 2.45 | 2.00 | 2.45 | 2.00 | 2.00 | 2.00 | 2.00 | 2.83 | 2.45 | **1.41** | 2.00 | 2.00 | 2.00 | 2.00 | 2.00 | 2.00 | 2.00 | 1.73 | 2.00 | 2.00 | 2.00 | 2.00 | 2.00 | 2.00 | 1.41 | 2.00 | 2.00 | 1.41 | 2.00 | 2.24 | 1.41 | 0.00 | 0.00 | 2.00 | 2.00 | 2.00 | 2.45 | 2.00 | 2.00 |
| P33 | 2.00 | 2.00 | 2.00 | 2.00 | 1.41 | 2.00 | 2.00 | 2.00 | 2.00 | 2.00 | 2.00 | 2.00 | 2.00 | 2.00 | 2.00 | 2.00 | 2.00 | **1.41** | 2.00 | 1.41 | 2.00 | 1.41 | 1.41 | 2.00 | 2.45 | 1.73 | 2.00 | 1.41 | 2.00 | 2.00 | 2.00 | 2.00 | 2.00 | 2.00 | 2.00 | 2.00 | 2.00 | 1.73 | 2.00 | 2.00 | 2.00 | 0.00 | 1.41 | 2.00 | 2.00 | 2.00 | 2.00 |
| P45 | 2.00 | 2.00 | 2.45 | 2.00 | 1.41 | 2.00 | 2.00 | 2.00 | 2.00 | 2.45 | 2.45 | 2.45 | 2.00 | 2.00 | 2.00 | 2.00 | 2.00 | **1.41** | 2.00 | 2.00 | 2.00 | 2.00 | 2.00 | 2.00 | 2.45 | 1.73 | 2.00 | 2.00 | 2.00 | 2.00 | 2.00 | 1.41 | 2.00 | 1.41 | 2.00 | 2.00 | 1.41 | 1.73 | 2.00 | 2.00 | 2.00 | 1.41 | 0.00 | 2.00 | 2.00 | 2.00 | 2.00 |
| P34 | 2.83 | 2.45 | 2.00 | 2.83 | 2.00 | 2.00 | 2.00 | 2.00 | 2.00 | 2.00 | 2.00 | 1.41 | 2.00 | 2.00 | 1.41 | 2.00 | 2.00 | **1.41** | 2.45 | 2.00 | 2.00 | 2.00 | 2.00 | 2.00 | 2.00 | 1.73 | 2.00 | 2.00 | 2.00 | 0.00 | 1.41 | 1.41 | 2.00 | 2.00 | 2.00 | 2.00 | 2.00 | 1.73 | 2.00 | 2.00 | 2.00 | 2.00 | 2.00 | 0.00 | 1.41 | 0.00 | 1.41 |
| P46 | 2.45 | 2.00 | 1.41 | 2.45 | 2.00 | 2.00 | 1.41 | 2.00 | 2.00 | 2.00 | 1.41 | 2.00 | 2.00 | 2.00 | 2.00 | 1.41 | 2.00 | **1.41** | 2.45 | 2.00 | 2.00 | 2.00 | 2.00 | 2.00 | 2.00 | 1.73 | 2.00 | 2.00 | 2.00 | 1.41 | 1.41 | 1.41 | 2.00 | 2.00 | 2.00 | 2.00 | 2.00 | 1.73 | 2.45 | 2.45 | 2.45 | 2.00 | 2.00 | 1.41 | 0.00 | 1.41 | 1.41 |
| P35 | 2.83 | 2.45 | 2.00 | 2.83 | 2.00 | 2.00 | 2.00 | 2.00 | 2.00 | 2.00 | 2.00 | 1.41 | 2.00 | 2.00 | 1.41 | 2.00 | 2.00 | **1.41** | 2.45 | 2.00 | 2.00 | 2.00 | 2.00 | 2.00 | 2.00 | 1.73 | 2.00 | 2.00 | 2.00 | 0.00 | 1.41 | 1.41 | 2.00 | 2.00 | 2.00 | 2.00 | 2.00 | 1.73 | 2.00 | 2.00 | 2.00 | 2.00 | 2.00 | 0.00 | 1.41 | 0.00 | 1.41 |
| P36 | 2.45 | 2.00 | 2.00 | 2.45 | 2.00 | 2.00 | 2.00 | 2.00 | 2.00 | 2.00 | 2.00 | 2.00 | 2.00 | 2.00 | 2.45 | 2.00 | 2.00 | **1.41** | 2.45 | 2.00 | 1.41 | 2.00 | 2.00 | 2.00 | 2.00 | 1.73 | 2.00 | 2.00 | 2.00 | 1.41 | 0.00 | 1.41 | 2.00 | 2.00 | 1.41 | 2.00 | 2.00 | 1.73 | 2.00 | 2.00 | 2.00 | 2.00 | 2.00 | 1.41 | 1.41 | 1.41 | 0.00 |

**Supplementary Table S7.** Shannon entropy of amino acid conservations over the unique T-ORF8 variants

| **Serial name** | **SE** | **Serial name** | **SE** | **Serial name** | **SE** |
| --- | --- | --- | --- | --- | --- |
| P10 | 0.957979735 | P24 | 0.963438 | P22 | 0.966983 |
| P12 | 0.957979735 | P34 | 0.963438 | P23 | 0.966983 |
| P16 | 0.957979735 | P35 | 0.963438 | P25 | 0.966983 |
| P42 | 0.957979735 | P45 | 0.963693 | P26 | 0.966983 |
| P29 | 0.957979735 | P17 | 0.965186 | P44 | 0.966983 |
| P31 | 0.957979735 | P39 | 0.965186 | P33 | 0.966983 |
| P32 | 0.957979735 | P19 | 0.965186 | P46 | 0.966983 |
| P2 | 0.961524341 | P20 | 0.965186 | P36 | 0.966983 |
| P37 | 0.961524341 | P5 | 0.966983 | P4 | 0.968986 |
| P9 | 0.961524341 | P6 | 0.966983 | P21 | 0.968986 |
| P18 | 0.961524341 | P38 | 0.966983 | P8 | 0.970821 |
| P27 | 0.961524341 | P11 | 0.966983 | P14 | 0.970821 |
| P28 | 0.961524341 | P13 | 0.966983 | P30 | 0.970821 |
| P43 | 0.961524341 | **P15** | **0.966983** | P1 | 0.972441 |
| P47 | 0.963438001 | P40 | 0.966983 | P7 | 0.972441 |
| P3 | 0.963438001 | P41 | 0.966983 |  |  |

**Supplementary Table S8**: Molecular and physicochemical information related to the T-ORF8 unique variants

| **Sl. Name** | **PI** | **EC** | **II** | **AI** | **GRAVY** | **PS** | **Tiny** | **Small** | **Aliphatic** | **Aromatic** | **Non-polar** | **Polar** | **Charged** | **Basic** | **Acidic** |
| --- | --- | --- | --- | --- | --- | --- | --- | --- | --- | --- | --- | --- | --- | --- | --- |
| **ORF8** | 5.42 | 16305 | 45.36 | 97.36 | 0.219 | 0.462 | 31 | 59 | 37 | 20 | 73 | 48 | 26 | 13 | 13 |
| **P1** | 6.68 | 125 | 85.17 | 90 | 0.731 | 0.553 | 10 | 12 | 8 | 5 | 15 | 11 | 4 | 3 | 1 |
| **P2** | 6.49 | 125 | 85.17 | 108.85 | 1.012 | 0.436 | 10 | 11 | 9 | 4 | 16 | 10 | 3 | 2 | 1 |
| **P3** | 6.49 | 125 | 85.17 | 90 | 0.962 | 0.553 | 10 | 12 | 8 | 5 | 16 | 10 | 3 | 2 | 1 |
| **P4** | 5.38 | 125 | 95.85 | 105 | 0.881 | 0.578 | 9 | 11 | 9 | 4 | 15 | 11 | 4 | 2 | 2 |
| **P5** | 6.49 | 125 | 85.17 | 108.85 | 1.096 | 0.492 | 10 | 12 | 10 | 4 | 17 | 9 | 3 | 2 | 1 |
| **P6** | 6.49 | 125 | 85.17 | 120 | 1.2 | 0.492 | 9 | 11 | 10 | 4 | 17 | 9 | 3 | 2 | 1 |
| **P7** | 7.84 | 125 | 81.91 | 105 | 0.877 | 0.578 | 9 | 11 | 9 | 4 | 16 | 10 | 4 | 3 | 1 |
| **P8** | 6.49 | 125 | 92.58 | 105 | 0.965 | 0.492 | 9 | 12 | 9 | 4 | 17 | 9 | 3 | 2 | 1 |
| **P9** | 6.49 | 125 | 85.17 | 108.85 | 1.012 | 0.436 | 10 | 11 | 9 | 4 | 16 | 10 | 3 | 2 | 1 |
| **P10** | 6.49 | 125 | 85.17 | 108.85 | 0.985 | 0.436 | 10 | 11 | 9 | 4 | 16 | 10 | 3 | 2 | 1 |
| **P11** | 6.49 | 125 | 68.27 | 105 | 1 | 0.492 | 10 | 13 | 9 | 4 | 16 | 10 | 3 | 2 | 1 |
| **P12** | 6.49 | 125 | 55.73 | 120 | 1.177 | 0.492 | 9 | 11 | 10 | 4 | 17 | 9 | 3 | 2 | 1 |
| **P13** | 6.49 | 125 | 72.63 | 120 | 1.2 | 0.492 | 9 | 11 | 10 | 4 | 17 | 9 | 3 | 2 | 1 |
| **P14** | 6.49 | 125 | 72.63 | 105 | 0.892 | 0.492 | 9 | 12 | 9 | 4 | 16 | 10 | 3 | 2 | 1 |
| **P15** | 6.49 | 125 | 85.17 | 105 | 1 | 0.492 | 10 | 12 | 9 | 4 | 16 | 10 | 3 | 2 | 1 |
| **P16** | 6.5 | 0 | 60.1 | 105 | 1.012 | 0.492 | 9 | 11 | 9 | 5 | 16 | 10 | 3 | 2 | 1 |
| **P17** | 8 | 0 | 67.5 | 105 | 0.731 | 0.544 | 9 | 11 | 9 | 4 | 15 | 11 | 4 | 3 | 1 |
| **P18** | 6.5 | 0 | 67.5 | 105 | 0.873 | 0.492 | 10 | 12 | 9 | 4 | 15 | 11 | 3 | 2 | 1 |
| **P19** | 8 | 0 | 85.17 | 105 | 0.731 | 0.544 | 9 | 11 | 9 | 4 | 15 | 11 | 4 | 3 | 1 |
| **P21** | 7.85 | 125 | 60.87 | 105 | 1.119 | 0.578 | 11 | 13 | 9 | 4 | 17 | 9 | 2 | 2 | 0 |
| **P22** | 7.82 | 125 | 92.58 | 105 | 0.95 | 0.597 | 10 | 12 | 9 | 3 | 16 | 10 | 3 | 2 | 1 |
| **P24** | 6.49 | 125 | 85.17 | 120 | 1.038 | 0.543 | 10 | 12 | 10 | 3 | 16 | 10 | 3 | 2 | 1 |
| **P25** | 6.49 | 125 | 85.17 | 105 | 0.862 | 0.543 | 11 | 13 | 9 | 3 | 15 | 11 | 3 | 2 | 1 |
| **P26** | 6.49 | 125 | 85.17 | 116.15 | 1.054 | 0.543 | 10 | 13 | 10 | 3 | 16 | 10 | 3 | 2 | 1 |
| **P27** | 6.49 | 125 | 85.17 | 112.31 | 1.092 | 0.492 | 9 | 12 | 9 | 4 | 16 | 10 | 3 | 2 | 1 |
| **P28** | 6.49 | 125 | 85.17 | 101.15 | 0.9 | 0.492 | 10 | 12 | 8 | 4 | 15 | 11 | 3 | 2 | 1 |
| **P29** | 6.49 | 125 | 81.91 | 101.15 | 0.904 | 0.492 | 10 | 12 | 8 | 4 | 15 | 11 | 3 | 2 | 1 |
| **P30** | 6.49 | 125 | 84.4 | 105 | 1.027 | 0.492 | 9 | 11 | 9 | 4 | 16 | 9 | 3 | 2 | 1 |
| **P31** | 6.49 | 125 | 85.17 | 90 | 0.8 | 0.492 | 11 | 13 | 8 | 4 | 15 | 11 | 3 | 2 | 1 |
| **P32** | 6.49 | 125 | 85.17 | 90 | 0.8 | 0.492 | 11 | 13 | 8 | 4 | 15 | 11 | 3 | 2 | 1 |
| **P33** | 7.85 | 125 | 95.85 | 105 | 0.842 | 0.544 | 9 | 11 | 9 | 4 | 15 | 11 | 4 | 3 | 1 |
| **P34** | 6.49 | 125 | 85.17 | 120 | 1.038 | 0.543 | 10 | 12 | 10 | 3 | 16 | 10 | 3 | 2 | 1 |
| **P35** | 6.49 | 125 | 81.91 | 120 | 1.038 | 0.543 | 10 | 12 | 10 | 3 | 16 | 10 | 3 | 2 | 1 |
| **P36** | 6.49 | 125 | 85.17 | 105 | 0.862 | 0.543 | 11 | 13 | 9 | 3 | 15 | 11 | 3 | 2 | 1 |
| **P37** | 6.49 | 125 | 85.17 | 97.69 | 0.908 | 0.436 | 11 | 12 | 9 | 4 | 16 | 10 | 3 | 2 | 1 |
| **P38** | 4.37 | 125 | 89.92 | 105 | 0.988 | 0.63 | 10 | 13 | 9 | 3 | 16 | 10 | 3 | 2 | 1 |
| **P39** | 7.85 | 125 | 80.04 | 105 | 0.962 | 0.544 | 10 | 12 | 9 | 4 | 16 | 10 | 4 | 3 | 1 |
| **P40** | 7.85 | 125 | 60.1 | 105 | 1.135 | 0.578 | 10 | 12 | 9 | 4 | 16 | 9 | 2 | 2 | 0 |
| **P41** | 5.75 | 125 | 82.27 | 105 | 0.988 | 0.545 | 10 | 13 | 9 | 3 | 16 | 10 | 2 | 1 | 1 |
| **P42** | 6.49 | 125 | 89.92 | 101.5 | 0.904 | 0.492 | 10 | 12 | 8 | 4 | 15 | 11 | 3 | 2 | 1 |
| **P43** | 6.49 | 125 | 85.17 | 112.31 | 1.092 | 0.492 | 9 | 12 | 9 | 4 | 16 | 10 | 3 | 2 | 1 |
| **P44** | 6.49 | 125 | 84.07 | 90 | 0.9 | 0.492 | 10 | 12 | 8 | 4 | 16 | 10 | 3 | 2 | 1 |
| **P45** | 6.49 | 125 | 88.44 | 116.15 | 1.177 | 0.492 | 9 | 12 | 10 | 4 | 16 | 10 | 3 | 2 | 1 |
| **P46** | 6.49 | 125 | 89.32 | 120 | 1.065 | 0.543 | 10 | 12 | 10 | 3 | 16 | 10 | 3 | 2 | 1 |
| **P47** | 6.49 | 125 | 85.17 | 90 | 0.962 | 0.553 | 10 | 12 | 8 | 5 | 16 | 10 | 3 | 2 | 1 |

**Supplementary Table S9.** Distance matrix

| Sl. Name | P15 | Sl. Name | P15 | Sl. Name | P15 | Sl. Name | P15 | Sl. Name | P15 |
| --- | --- | --- | --- | --- | --- | --- | --- | --- | --- |
| P15 | 0 | P5 | 4.222762 | P22 | 7.595428 | P3 | 15.06669 | P11 | 16.92956 |
| P30 | 1.895687 | P28 | 4.222854 | P8 | 7.609818 | P47 | 15.06669 | P13 | 19.67973 |
| P25 | 2.240903 | P29 | 5.334727 | P4 | 11.01436 | P44 | 15.07382 | P21 | 24.46142 |
| P36 | 2.240903 | P38 | 5.392225 | P33 | 11.04263 | P6 | 15.16707 | P40 | 25.16704 |
| P41 | 3.600077 | P39 | 5.492781 | P26 | 11.28397 | P31 | 15.16707 | P12 | 33.11714 |
| P2 | 3.978163 | P42 | 6.149936 | P45 | 11.7067 | P32 | 15.16707 | P19 | 125.0334 |
| P9 | 3.978163 | P27 | 7.378656 | P14 | 12.58027 | P1 | 15.20237 | P18 | 126.2507 |
| P10 | 3.978173 | P43 | 7.378656 | P24 | 15.06665 | P35 | 15.41531 | P17 | 126.2758 |
| P7 | 4.058648 | P37 | 7.378868 | P34 | 15.06665 | P46 | 15.62784 | P16 | 127.501 |

**Supplementary Table S10.** Distance from the P15 variant to the unique T-ORF8 variants, based on the physicochemical feature vectors

| **Sl. Name** | P15 | **Sl. Name** | P15 | **Sl. Name** | P15 | **Sl. Name** | P15 | **Sl. Name** | P15 |
| --- | --- | --- | --- | --- | --- | --- | --- | --- | --- |
| **P15** | 0 | **P5** | 4.222762 | **P22** | 7.595428 | **P3** | 15.06669 | **P11** | 16.92956 |
| **P30** | 1.895687 | **P28** | 4.222854 | **P8** | 7.609818 | **P47** | 15.06669 | **P13** | 19.67973 |
| **P25** | 2.240903 | **P29** | 5.334727 | **P4** | 11.01436 | **P44** | 15.07382 | **P21** | 24.46142 |
| **P36** | 2.240903 | **P38** | 5.392225 | **P33** | 11.04263 | **P6** | 15.16707 | **P40** | 25.16704 |
| **P41** | 3.600077 | **P39** | 5.492781 | **P26** | 11.28397 | **P31** | 15.16707 | **P12** | 33.11714 |
| **P2** | 3.978163 | **P42** | 6.149936 | **P45** | 11.7067 | **P32** | 15.16707 | **P19** | 125.0334 |
| **P9** | 3.978163 | **P27** | 7.378656 | **P14** | 12.58027 | **P1** | 15.20237 | **P18** | 126.2507 |
| **P10** | 3.978173 | **P43** | 7.378656 | **P24** | 15.06665 | **P35** | 15.41531 | **P17** | 126.2758 |
| **P7** | 4.058648 | **P37** | 7.378868 | **P34** | 15.06665 | **P46** | 15.62784 | **P16** | 127.501 |
